# Subcytoplasmic location of translation controls protein output

**DOI:** 10.1016/j.molcel.2023.11.025

**Published:** 2023-12-21

**Authors:** Ellen L. Horste, Mervin M. Fansler, Ting Cai, Xiuzhen Chen, Sibylle Mitschka, Gang Zhen, Flora C.Y. Lee, Jernej Ule, Christine Mayr

**Affiliations:** 1 Gerstner Sloan Kettering Graduate School of Biomedical Sciences, New York, NY 10065, USA; 2 Cancer Biology and Genetics Program, Sloan Kettering Institute, New York, NY 10065, USA; 3 Tri-Institutional Training Program in Computational Biology and Medicine, Weill-Cornell Graduate College, New York, NY 10021, USA; 4 UK Dementia Research Institute, King’s College London, London SE5 9NU, UK; 5 The Francis Crick Institute, 1 Midland Road, London NW1 1AT, UK; 6 Lead contact

## Abstract

The cytoplasm is highly compartmentalized, but the extent and consequences of subcytoplasmic mRNA localization in non-polarized cells are largely unknown. We determined mRNA enrichment in TIS granules (TGs) and the rough endoplasmic reticulum (ER) through particle sorting and isolated cytosolic mRNAs by digitonin extraction. When focusing on genes that encode non-membrane proteins, we observed that 52% have transcripts enriched in specific compartments. Compartment enrichment correlates with a combinatorial code based on mRNA length, exon length, and 3′ UTR-bound RNA-binding proteins. Compartment-biased mRNAs differ in the functional classes of their encoded proteins: TG-enriched mRNAs encode low-abundance proteins with strong enrichment of transcription factors, whereas ER-enriched mRNAs encode large and highly expressed proteins. Compartment localization is an important determinant of mRNA and protein abundance, which is supported by reporter experiments showing that redirecting cytosolic mRNAs to the ER increases their protein expression. In summary, the cytoplasm is functionally compartmentalized by local translation environments.

## In brief

Horste et al. find that mRNA transcripts that encode non-membrane proteins are not evenly distributed across the cytoplasm. Instead, functionally related groups of transcripts are enriched in compartments that are not separated by membranes. The localization pattern correlates with gene architecture features and the binding pattern of 3′ UTR-bound RNA-binding proteins.

## Graphical abstract

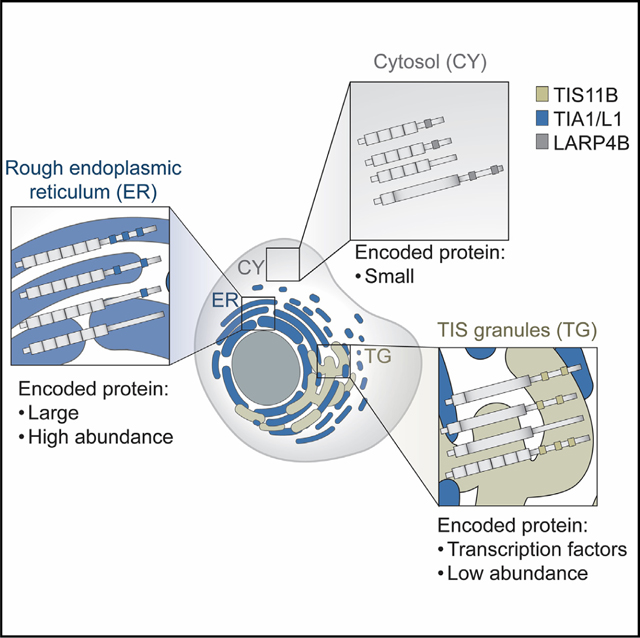

## INTRODUCTION

In polarized cells such as neurons, intestinal epithelial cells, or cells of the early fly embryo, the majority of mRNAs have a distinct spatial localization pattern.^1–5^ mRNA localization enables the local control of protein production.^6–8^ In non-polarized cells, mRNA localization has primarily been studied for membrane proteins.^9–12^ Whereas the rough endoplasmic reticulum (ER) is established as a major site of local protein synthesis for membrane and secretory proteins,^9,10,13^ the cytoplasm is compartmentalized by additional membrane-bound and membraneless organelles.^14–17^ These compartments may enable the generation of unique biochemical translation environments, which have been suggested to be crucial for protein interaction partner selection during protein synthesis.^16,18–20^ However, it is currently largely unknown whether the location of protein synthesis also matters for protein output.

TIS granules (TGs) represent one such unique translation compartment, which promotes the co-translational formation of protein complexes. Both endogenous and overexpressed TIS11B promote the formation of specific protein complexes when mRNAs are translated in TGs.^16,19^ TGs are formed by the RNA-binding protein (RBP) TIS11B, together with its bound mRNAs.^16,21^ *TIS11B* mRNA is ubiquitously expressed,^22^ suggesting that TGs are widespread. TGs are present under steady-state cultivation conditions and form a network-like structure that is intertwined with the rough ER.^16,21^ To investigate the broader biological significance of TGs, we determined the mRNAs enriched in TGs, the neighboring rough ER, and the surrounding cytosol.

**Figure 1.**
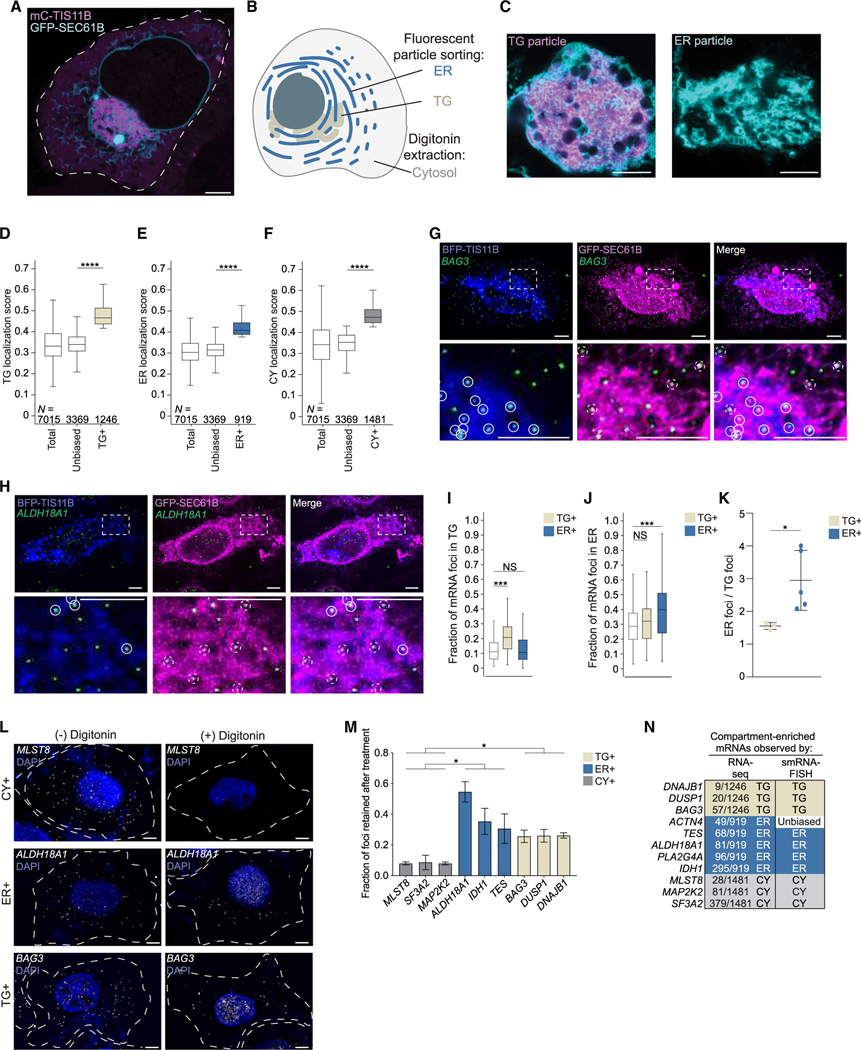
Strategy to identify compartment-enriched mRNAs (A) Confocal live cell imaging of HeLa cells after transfection of mCherry (mC)-TIS11B and GFP-SEC61B to visualize TGs and the rough ER. Scale bars, 5 μm. (B) Schematic of a cell with three cytoplasmic compartments. (C) As in (A) but showing fluorescent TG (left) and ER (right) particles. (D) Transcript localization scores obtained from TG samples. Mann-Whitney test, p = 0. Boxplots depict median, 25th and 75th percentiles (box), and 5% and 95% confidence intervals (error bars). (E) Transcript localization scores obtained from ER samples. Mann-Whitney test, p = 1 × 10^−123^. (F) Transcript localization scores obtained from CY samples. Mann-Whitney test, p = 0. (G) smRNA-FISH of endogenous TG+ mRNA *BAG3* (green) in HeLa cells. TIS granules (BFP-TIS11B, blue) and the ER (GFP-SEC61B, magenta) were simultaneously visualized. Bottom panel shows 5 × zoom-in of boxed area. White circles: mRNA colocalization with TG, dashed white circles: mRNA colocalization with ER. Representative images are shown. Scale bars, 5 μm. (H) As in (G), but smRNA-FISH of the ER+ mRNA *ALDH18A1*. (I) Quantification of smRNA-FISH foci. White boxplot: expected fraction of mRNA transcripts based on TG compartment size (n = 186 cells). ***p = 5 × 10^−11^ (Mann-Whitney test). Additional images in Figures S2A–S2F. Individual values are shown in Figures S2H and S2I. (J) As in (I). White boxplot: expected fraction of mRNA transcripts based on ER compartment size (n = 186 cells). ***p = 1 × 10^−6^. (K) The ratio of smRNA-FISH foci colocalizing with the ER compared with the foci colocalizing with TGs, shown for mRNAs from (I) and (J). t test for independent samples, *p = 0.044. Horizontal line, median; error bars; 25th and 75th percentiles. (L) smRNA-FISH foci of endogenous mRNAs in HeLa cells before (−) and after (+) digitonin extraction. Dotted lines indicate cell boundaries. Representative images are shown. Scale bars, 5 μm. (M) Quantification of (L). Shown is the fraction of digitonin-resistant smRNA-FISH foci of endogenous mRNAs as mean ± SD of three independent experiments. Number of cells analyzed, see Table S2. Additional images in Figures S3A–S3C. t test for independent samples, *p < 0.041. (N) smRNA-FISH validation summary. Shown is ranking obtained from localization scores.

Because TIS11B protein is present in cells in two states (Figure 1A), as soluble cytosolic protein and as phase-separated TG network,^16,21^ we decided to use fluorescent particle sorting^23^ to identify TG-enriched mRNAs. We also applied fluorescent particle sorting to isolate ER-enriched mRNAs and extracted cytosolic mRNAs using digitonin. We analyzed genes that encode non-membrane proteins and found more than 3,600 that have transcripts enriched in one of the three compartments. mRNAs enriched in each compartment share similar mRNA architectures, which differ strongly between compartments. Compartment-enriched mRNAs also differed significantly in production and degradation rates as well as in the functional classes and expression levels of their encoded proteins. TIS11B knockout (KO) and reporter experiments support a model by which a combinatorial code based on mRNA architecture features, together with 3′ UTR-bound RBPs TIS11B, TIA1/ L1, and LARP4B, correlated with the compartment-biased mRNA localization pattern. Intriguingly, we observed that redirecting cytosolic mRNAs to the ER controls protein expression, which indicates that protein abundance is regulated by the location of translation in the cytoplasm.

## RESULTS

### Approach to determine subcytoplasmic mRNA localization

We set out to identify mRNAs that are localized in non-polarized human HEK293T cells under steady-state cultivation conditions. We focused on three major unenclosed cytoplasmic compartments−TGs, a condensate network formed by the RBP TIS11B, the cytosolic surface of the ER, and the soluble part of the cytoplasm known as the cytosol (Figure 1B). For simplicity, we consider here the sum of the three compartments as the universe of cytoplasmic mRNAs.

To identify TG-enriched (TG+) and ER-enriched (ER+) mRNAs, we performed fluorescent particle sorting followed by RNA sequencing (RNA-seq). To label TGs and rough ER, we co-transfected cells with mCherry-TIS11B and GFP-SEC61B, respectively. After flow-cytometry-based sorting of fluorescent particles, we used confocal microscopy and western blot analysis to assess the purity of the particles (Figures S1A–S1E). We modestly overexpressed mCherry-TIS11B compared with its endogenous levels (Figure S1C), which resulted in approximately 30% of cells forming TGs. This amount was chosen because 25%–30% of HEK293T cells form TGs from endogenous TIS11B. ER particles did not contain mCherry-TIS11B. TG particles contained GFP-SEC61B, but they contained 13-fold more mCherry-TIS11B than ER particles (Figures 1C and S1C–S1E). As TGs are defined by the presence of TIS11B,^16^ we reasoned that the strong overrepresentation of TIS11B in TG particles would allow us to identify relative enrichments of mRNAs between the compartments.

To isolate cytosolic mRNAs, we used digitonin extraction.^24^ The extracted cytosol was not contaminated by nuclei or the ER, but it contained TIS11B, which was expected because soluble TIS11B is known to be present in the cytosol (Figure S1C). We performed RNA-seq on biological replicate samples to determine the mRNA composition in the three fractions and focused our analysis on protein-coding mRNAs (Figure S1F).

### mRNAs that encode membrane or secretory proteins largely localize to the ER membrane

We investigated whether the relative mRNA transcript distribution differs across the three compartments. For each gene, we determined a compartment-specific localization score (LS). This score is calculated using the reads per kilobase per million mapped reads (RPKM) value obtained in each of the three compartments, respectively, and dividing it by the sum of the RPKM values in all three compartments. Thus, each gene is assigned three LSs that correspond to the fraction of its transcripts localizing to each of the three compartments: TGs, the ER, and the cytosol.

First, we focused on mRNAs that encode membrane or secretory proteins, which are known to be translated on the ER.^10,11,13^ In line with previous analyses, we find preferential partitioning of mRNAs encoding membrane/secretory proteins in the ER samples (Figure S1G).^10,11,13^ To validate our compartment isolation method, we compared it with datasets from three alternative isolation methods.^9,11,13^ We consider 69% (N = 1,476) of membrane/secretory proteins to be enriched on the ER (Figure S1H; Table S1) and we detected between 80% and 90% overlap between our data and previous methods (Figures S1I and S1J).^9,11,13^ These results strongly support the validity of our purification strategy for mRNAs that encode membrane/secretory proteins.

### Half of the genes that encode non-membrane proteins have a biased cytoplasmic transcript distribution

For mRNAs that encode non-membrane proteins, we observed that their LSs were more evenly distributed across the compartments (Figure S1G). To identify absolute differences in mRNA distribution, the relative size of each compartment needs to be considered. However, this parameter is currently unknown.

Therefore, we instead calculated the relative enrichment of mRNAs within each compartment. We considered an mRNA to be compartment-enriched if its mean LS across biological replicates was at least 1.25-fold higher than the median LS of the compartment samples (Figures 1D–1F). Based on this criterion, we identified 1,246 TG+ mRNAs, 919 ER+ mRNAs, and 1,481 mRNAs enriched in the cytosol (CY+), which were non-overlapping (Figures 1D–1F; Table S1). The remaining 3,369 mRNAs were not enriched in a single compartment and were considered to have an unbiased localization pattern (Figures 1D–1F and S1K). The distribution of LSs of TG+, ER+, or CY+ mRNAs is significantly different from the LSs of mRNAs with unbiased localization patterns (Figures 1D–1F). Because LSs across the compartments sum to 1, an mRNA enriched in one compartment is relatively de-enriched in the other two (Figure S1L). Based on this strategy, 52% of genes that encode non-membrane proteins have transcripts that are significantly enriched in one of the three subcytoplasmic compartments in steady-state conditions.

As a recent study also analyzed the relative distribution of mRNA transcripts across subcellular compartments, we compared our data with their results.^25^ Although their dataset was generated by density gradient centrifugation in a different cell line, the two datasets strongly agreed in a qualitative and quantitative manner (Figure S1M), suggesting that our isolation method as well as our strategy to define compartment-enriched mRNAs are valid. As non-membrane protein-encoding mRNAs with biased transcript distributions in the cytoplasm have not been systematically characterized, we focused all subsequent analyses on mRNAs that encode non-membrane proteins.

### Validation of compartment-enriched mRNAs by smRNA-FISH

We further validated the mRNAs designated as compartment-enriched by performing single-molecule RNA-fluorescence *in situ* hybridization (smRNA-FISH) on endogenous mRNAs.^26^ Candidates for validation were primarily chosen based on their respective LSs, and most ranked in the top 10% of their respective compartments (Table S2). To distinguish between TG+ and ER+ mRNAs, we performed smRNA-FISH together with co-transfection of blue fluorescent protein (BFP)-TIS11B and GFP-SEC61B to simultaneously visualize mRNA puncta, TGs, and the rough ER (Figures 1G, 1H, and S2A–S2G). We considered an mRNA to have an unbiased localization pattern if its transcript distribution correlated with compartment size. As proxy for relative compartment size, we used the areas of the maximum projection of the fluorescent signals for each compartment and compared them to the whole-cell area. For unbiased mRNAs, we expect that 11% of transcripts localize to TGs and 29% of transcripts localize to the ER (Figures 1I and 1J).

For 3/3 TG+ mRNAs, we observed a significant enrichment of mRNA puncta in TGs but not on the ER (Figures 1G–1J, S2A, S2B, S2H, and S2I). For the five ER+ mRNAs tested, the mRNA puncta of 4/5 mRNAs were significantly enriched on the ER and, for all five, we observed a 2- to 4-fold higher fraction of mRNA puncta that colocalized with the ER compared with TGs (Figures 1H–1K and S2C–S2I).

Cytosolic mRNAs were isolated through digitonin extraction. This means that CY+ mRNAs localize to the soluble part of the cytoplasm and are not attached to cytoplasmic structures, including membranes or the cytoskeleton. As smRNA-FISH only informs on co-localization and not attachment, we validated CY+ mRNAs by performing smRNA-FISH before and after digitonin extraction and calculated the fraction of retained mRNAs. Both TG+ and ER+ mRNAs showed more retention than CY+ mRNAs, which were depleted by about 90% following digitonin treatment (Figures 1L, 1M, and S3A–S3C). This confirms that CY+ mRNAs predominantly localize to the soluble part of the cytoplasm. Taken together, as we successfully validated 10/11 mRNAs that were designated to be TG+ or ER+ or CY+ (Figure 1N), we conclude that about half (52%) of genes that encode non-membrane proteins have transcripts that are enriched in distinct subcytoplasmic compartments.

### mRNA and protein levels strongly correlate with the location of translation

**Figure 2.**
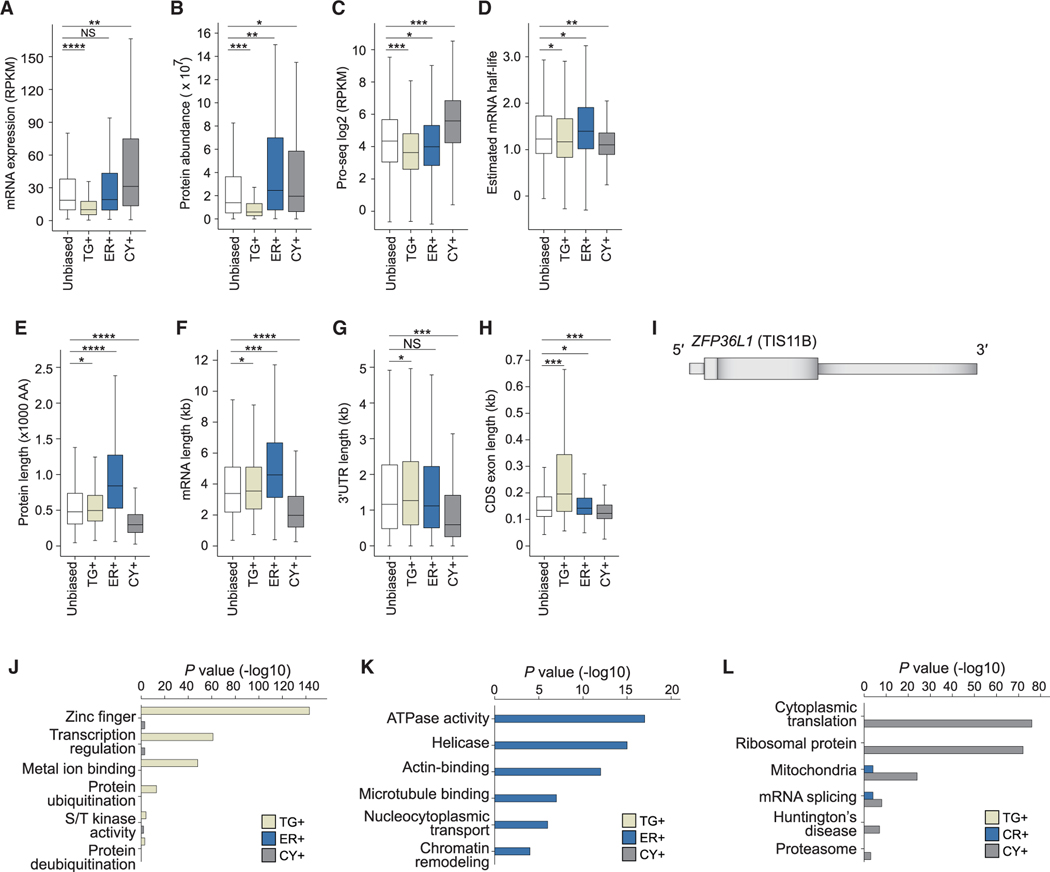
Characteristics of compartment-enriched mRNAs (A) Steady-state mRNA abundance levels obtained from whole-cell lysates. TG+, N = 1,246; ER+, N = 919, CY+, N = 1,481; unbiased, N = 3,369. Mann-Whitney test: *0.05 > p > 10^−9^; **10^−10^ >p> 10^−20^; ***10^−21^ >p> 10^−80^; ****10^−81^ > p > 0. Exact p values are listed in Table S3. Boxplots depict median, 25th and 75th percentiles (box), and 5% and 95% confidence intervals (error bars). (B) As in (A), but steady-state protein levels obtained from whole-cell lysates are shown. TG+, N = 469; ER+, N = 638; CY+, N = 833; unbiased, N = 2,001. (C) As in (B), but Pro-seq levels are shown, which indicate transcription rates. TG+, N = 1,222; ER+, N = 896; CY+, N = 1,425; unbiased, N = 3,268. (D) As in (C), but estimated mRNA half-lives are shown. (E) As in (A), but protein size distributions are shown. AA, amino acid. (F) As in (A), but mRNA length distributions are shown. (G) As in (A), but 3′ UTR length distributions are shown. (H) As in (A), but average CDS exon length distributions are shown. (I) ZFP36L1 (TIS11B) mRNA model. Tall boxes: CDS exons, narrow boxes: 5′ and 3′ UTRs. (J) Gene ontology analysis for TG+ mRNAs. Top six functional gene classes uniquely enriched in TG+ mRNAs and Benjamini Hochberg-adjusted p values are shown. (K) As in (J), but for ER+ mRNAs. (L) As in (J), but for CY+ mRNAs.

Next, we characterized the features of compartment-enriched mRNAs and found substantial differences in their steady-state mRNA and protein levels (Figures 2A, 2B, S4A, and S4B). TG+ mRNAs have the lowest steady-state expression levels and encode proteins with the lowest expression levels (Figures 2A and 2B). To examine whether the low mRNA levels are caused by high mRNA degradation rates, we estimated mRNA half-lives by analyzing precision run-on sequencing (Pro-seq) and RNA-seq data (Figures 2C, 2D, and S4C–S4E).^27,28^ Pro-seq values can be treated as transcription rates and RNA-seq data can be viewed as a measure of RNA concentration to estimate RNA decay rates required for a steady-state equilibrium.^28^ For TG+ mRNAs, we observed that their low steady-state levels were not primarily caused by a low mRNA stability. Instead, these mRNAs had the lowest transcription rates, suggesting that they are either produced at a low rate or have high cotranscriptional degradation rates (Figures 2C, 2D, S4D, and S4E).^29^ CY+ mRNAs had the highest degree of mRNA turnover, with both high production and degradation rates (Figures 2C and 2D). ER+ mRNAs encode proteins with the highest expression levels, particularly when normalizing to their intermediate steady-state mRNA levels (Figures 2A and 2B).

We further observed that the compartment-enriched mRNAs differed substantially in their gene architectures (Figures 2E–2H and S4F–S4K). ER+ mRNAs encode the largest proteins, with a median size of 840 amino acids−nearly 3 times larger than proteins encoded by CY+ mRNAs (Figure 2E). The difference in protein size was reflected in the large differences in exon number and mRNA length between ER+ and CY+ mRNAs (Figures 2F, S4J, and S4K). The median length of ER+ mRNAs is 4,600 nucleotides (nt), whereas the median length of CY+ mRNAs is 2,000 nt. It is not surprising that CY+ mRNAs have the shortest 3′ UTRs (Figure 2G). TG+ mRNAs are uniquely characterized by large coding sequence (CDS) exons, with a median size of 200 nt compared with 133 nt for the remaining mRNAs (Figure 2H). Further analysis revealed that the majority of TG+ mRNAs have gene architectures similar to *ZFP36L1* (encoding TIS11B), which is characterized by a short first exon and a long last exon that contains ~95% of its CDS (Figure 2I).

Moreover, compartment-enriched mRNAs encode substantially different functional gene classes.^30^ Consistent with the low protein expression levels, TG+ mRNAs were strongly enriched in proteins containing zinc fingers and transcription factors, which are known to have low expression (Figure 2J).^31^ In contrast, ER+ mRNAs encode large and highly abundant proteins, such as cytoskeleton-binding proteins and chromatin regulators (Figure 2K). CY+ mRNAs often encode smaller proteins involved in the regulation of translation or splicing (Figure 2L).

### TGs support active translation

TGs may constitute a specialized translation environment for nuclear proteins that require low expression levels (Figures 2A, 2B, and 2J).^31^ For evidence of active translation, we used the SunTag system to visualize mRNAs and their nascent proteins (Figures S3D and S3E).^32^ We confirmed that TGs represent a translation environment, but the number of mRNA foci in TGs was 5-fold lower compared with the cytosol.^16,19^ As the proportion of mRNA translated was similar in TGs and the cytosol (Figures S3F and S3G), our data show that TGs are sites of active translation and that the low expression level of TG-translated proteins is predominantly a result of their low nuclear gene expression (Figures 2A and 2C).

### Differential 3′ UTR binding of RBPs correlates with compartment enrichment of mRNAs

**Figure 3.**
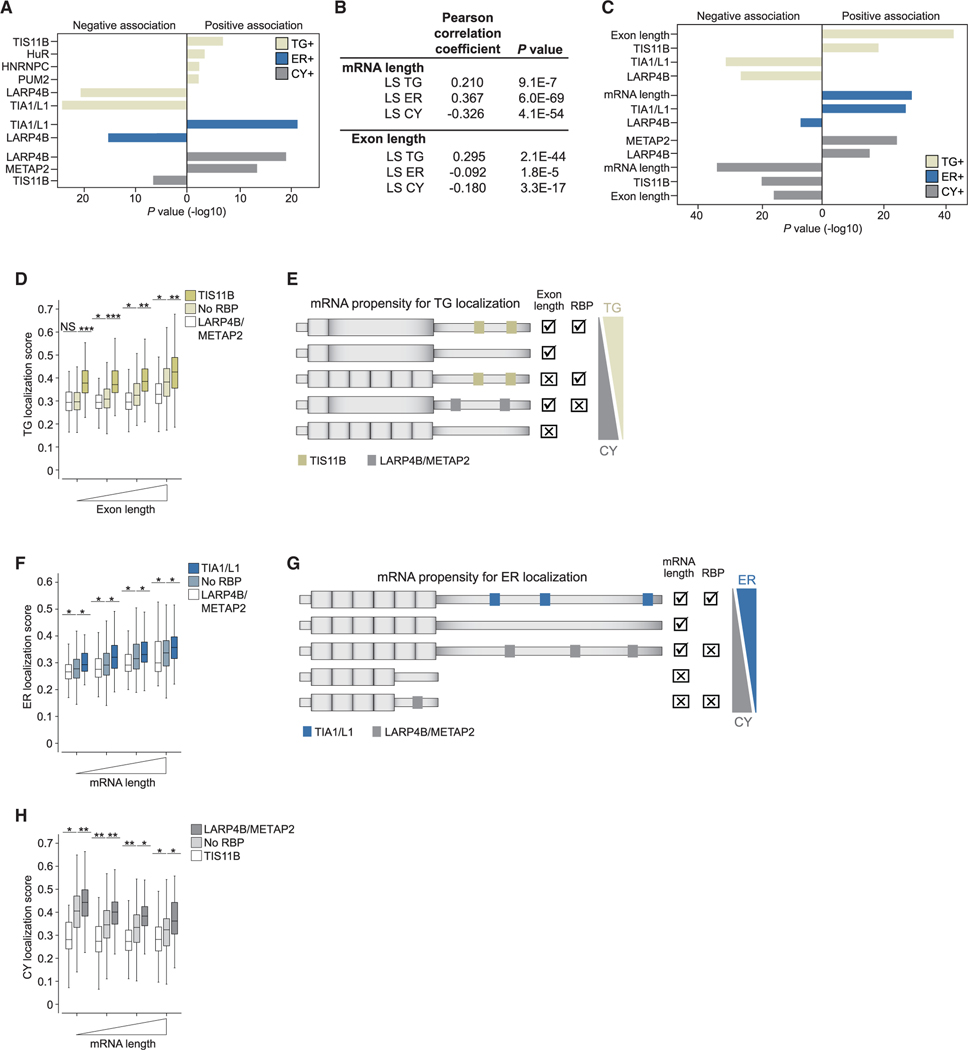
mRNA architecture features together with RBPs determine the subcytoplasmic transcript distribution (A) Logistic regression results for 3′ UTR-bound RBPs positively or negatively associated with compartment-enriched mRNAs. Full values in Table S4. (B) Pearson’s correlation coefficients of mRNA and coding exon length with compartment localization scores (LSs). (C) As in (A) but integrating 3′ UTR-bound RBPs from (A) and mRNA architecture features. (D) Propensity of mRNAs for TG localization stratified by coding exon length and bound RBPs. No RBP (N = 1,498), bound by LARP4B or METAP2 (N = 717) or by TIS11B (N = 834). Mann-Whitney test p values as Figure 2A. Boxplots depict median, 25th and 75th percentiles (box), and 5% and 95% confidence intervals (error bars). (E) Model showing additive effects of coding exon length and RBPs on mRNA localization propensity to TGs or the cytosol. Positive effect: (check), negative effect: (x) shown as in Figure 2I. (F) As in (D) for mRNA localization to the ER, stratified by mRNA length and bound RBPs. Bound by TIA1/L1 (N = 634). (G) As in (E) showing additive effects of mRNA length and RBPs on the mRNA localization propensity. (H) As in (D) for mRNAs localization to cytosol, stratified by mRNA length and bound RBPs.

Next, we identified the RBPs responsible for compartment enrichment of mRNA (Figures 1D–1F). As TIS11B is the scaffold protein of TGs,^16^ we performed used individual-nucleotide resolution UV-cross-linking and immunoprecipitation (iCLIP) of TIS11B in HEK293T cells (Figures S5A and S5B). We confirmed that the top binding motif of TIS11B in 3′ UTRs of mRNAs is the canonical AU-rich element (UAUUUA) (Figure S5C). We analyzed additional CLIP datasets to perform a comprehensive analysis on localization regulators.^33,34^ We found that 24/170 tested RBPs showed binding site enrichment in 3′ UTRs of compartment-enriched mRNAs (Table S4). Through logistic regression, we identified seven RBPs whose binding contributed most significantly to mRNA enrichment in the three compartments. They include TIS11B, HuR, PUM2, HNRNPC, TIA1/L1, LARP4B, and METAP2 (Figure 3A). As a previous CLIP analysis showed that peaks for TIA1 and TIAL1 cannot be distinguished,^35^ we used the sum of peaks from TIA1 and TIAL1 to obtain the values for TIA1/ L1. The presence of TIS11B, HuR, PUM2, and HNRNPC on mRNAs correlates with TG enrichment, TIA1/L1 correlates with ER enrichment, and LARP4B or METAP2 correlates with cytosol enrichment (Figure 3A).

### mRNA architecture features, together with RBPs, generate a combinatorial code for subcytoplasmic mRNA localization

As 2,154 mRNAs (30.7%) that encode non-membrane proteins were not bound by any of the seven RBPs (Figure S5D), we considered additional regulatory factors. Among these mRNAs, mRNA length correlated strongly with the ER and CY LSs, but in opposite directions, suggesting that long mRNAs associate with the ER (Figure 3B). Similarly, average CDS exon length correlated strongly, and in a positive manner, with the TG LS but negatively with the CY LS (Figure 3B).

Including mRNA and CDS exon length in the logistic regression identified mRNA architecture features, together with the presence of 3′ UTR-bound RBPs, as strong factors for compartment enrichment of mRNAs (Figure 3C; Table S4). To learn the rules for mRNA localization to the compartments, we plotted the propensity for TG enrichment and integrated the bound RBPs together with CDS exon length (Figure 3D). Binding of LARP4B/METAP2 always decreased, whereas binding of TIS11B or long CDS exons strongly increased the propensity of mRNA to localize to TGs. TIS11B and CDS exon length have additive effects, as mRNAs with both features showed the strongest TG enrichment (Figures 3D and 3E).

The two features that correlate best with mRNA localization to the ER are mRNA length and 3′ UTR-bound TIA1/L1. mRNAs that combine both features have the strongest propensity for ER localization (Figures 3F and 3G). In contrast, shorter mRNAs not bound by any RBP or bound by LARP4B/METAP2 tend to localize to the cytosol (Figures 3G and 3H). Taken together, our data suggest that subcytoplasmic mRNA localization is determined by a combinatorial code that integrates mRNA and exon length with the presence of RBPs (Figures 3E and 3G).

### TIS11B deletion changes subcytoplasmic mRNA transcript distribution

**Figure 4.**
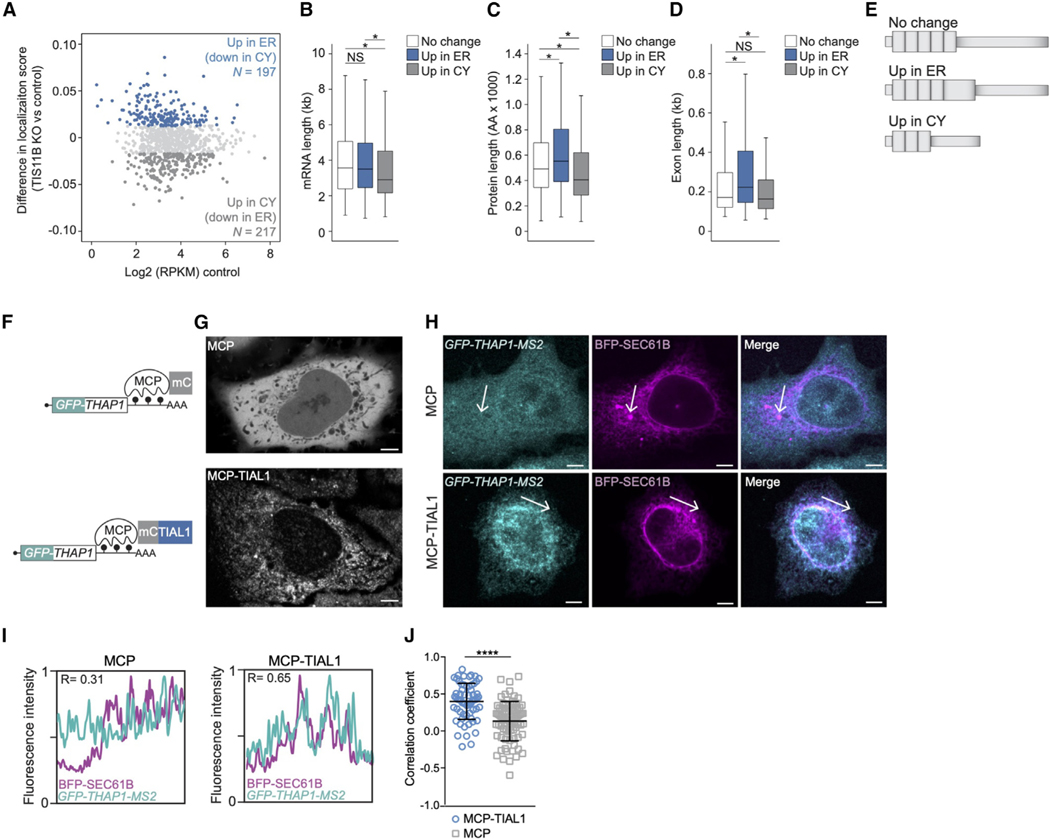
Experimental validation of regulators of subcytoplasmic mRNA transcript distribution (A) TG+ mRNAs are shown and are color-coded based on their change in compartment localization. No change (N = 508). (B) Length distribution of mRNAs from (A). Mann-Whitney test p values as in Figure 2A. (C) As in (B) but for protein size distribution. Boxplots depict median, 25th and 75th percentiles (box), and 5% and 95% confidence intervals (error bars). (D) As in (B) but for CDS exon length distribution. (E) As in Figure 3E but for mRNA features of TG+ mRNAs that change their localization upon TIS11B KO. (F) Schematic of mRNA reporter for validation of a 3′ UTR-bound RBP on mRNA localization. The GFP-THAP1 reporter mRNA contains MS2 hairpins as 3′ UTR, which bind to cotransfected MS2 coat protein (mCherry-tagged MCP). TIAL1-MCP fusion tethers TIAL1 to the reporter 3′ UTR. mC, mCherry. (G) Confocal live cell imaging of HeLa cells expressing the indicated constructs. Scale bars, 5 μm. (H) RNA-FISH (teal) of the GFP reporter mRNA from (F) in HeLa cells. GFP-SEC61B visualizes the rough ER (magenta). Representative images are shown. Scale bars, 5 μm. (I) Pearson’s correlation coefficients of fluorescence intensities at arrows in (H). (J) Quantification of (H) and (I). MCP (n = 26 cells), MCP-TIAL1 (n = 21). Horizontal line: median, error bars: 25th, 75th percentiles. Mann-Whitney test, ****p < 0.0001.

To experimentally test the proposed mRNA localization code, we generated HEK293T cells with an inducible KO of TIS11B, isolated ER particles, and extracted the cytosol (Figures S5E and S5F; Table S5). To examine where mRNAs designated as TG+ localize in the absence of TGs, we identified the top 20% of mRNA localization changes to the ER and the cytosol and intersected them with mRNAs designated as TG+ (Figure 4A). As only two compartments were isolated, increased mRNA localization to the ER means decreased cytosolic localization and vice versa (Figure 4A).

We did not find specific RBPs associated with the localization-changing mRNAs because TG+ mRNAs are mostly bound by TIS11B and only a few (13% and 15%) are LARP4B or TIA1/L1 targets (Table S5). However, the TG+ mRNAs that increased their cytosolic localization upon TIS11B KO were the shortest, encoded the smallest proteins, and had the shortest exon length (Figures 4B–4E). In contrast, TG+ mRNAs that increased their ER localization upon deletion of TIS11B were significantly longer, encoded the largest proteins, and had longer exons (Figure 4B–4E).

These results converge on a model where features that correlate with mRNA architecture set up a ‘‘default’’ steady-state pattern of mRNA transcript distribution, which can be overcome or reinforced through the binding of RBPs. Our model is consistent with the following observations: short mRNAs with average exon length localize to TGs when bound by TIS11B, but in the absence of TIS11B they revert to the transcript distribution established by mRNA architecture and the remaining bound RBPs, in this case the cytosol (Figure 3E). Similarly, longer TG+ mRNAs that encode the largest proteins localize to the ER upon loss of TIS11B (Figure 3G). Currently, the ‘‘readers’’ of the mRNA architecture features are unknown.

### 3′ UTR-bound TIAL1 promotes localization of non-membrane protein-encoding mRNAs to the ER

We set out to investigate the influence of TIA1/L1 on mRNA localization to the ER using TIA1/L1 double KO cells.^36^ However, as reported, these cells showed a high rate of cell death, which prevented us from obtaining high-quality particles. To validate TIA1/ L1-dependent mRNA localization to the ER, we used the MS2 tethering system to mimic 3′ UTR-binding of TIA1/L1 (Figure 4F). We generated a *GFP-THAP1* reporter mRNA that contained MS2-binding sites as 3′ UTR.^37–39^ Coexpression of mCherry-tagged MS2 coat protein (MCP) fused to TIAL1 tethers TIAL1 to the 3′ UTR of the reporter mRNA (Figure 4F). As a control, mCherry-tagged MCP was tethered.

Coexpression of the reporter mRNA and MCP evenly distributed both MCP protein and reporter mRNA in the cytosol (Figures 4F–4H). In contrast, coexpression of the reporter mRNA and MCP-TIAL1 resulted in perinuclear, reticulated expression of MCP-TIAL1, with the mRNA reporter predominantly localizing to the rough ER (Figures 4F–4H). Colocalization was assessed by RNA-FISH of the GFP-tagged reporter mRNA and simultaneous visualization of the rough ER through fluorescently tagged SEC61B. We quantified the overlap between the reporter mRNAs and the ER (Figure 4I). In the presence of MCP-TIAL1, we observed higher correlation coefficients between the fluorescence intensities (Figure 4J). This result indicated that 3′ UTR-bound TIAL1 was sufficient to induce localization of non-membrane protein encoding mRNAs to the rough ER surface.

### 3′ UTR-bound TIAL1 increases protein expression

**Figure 5.**
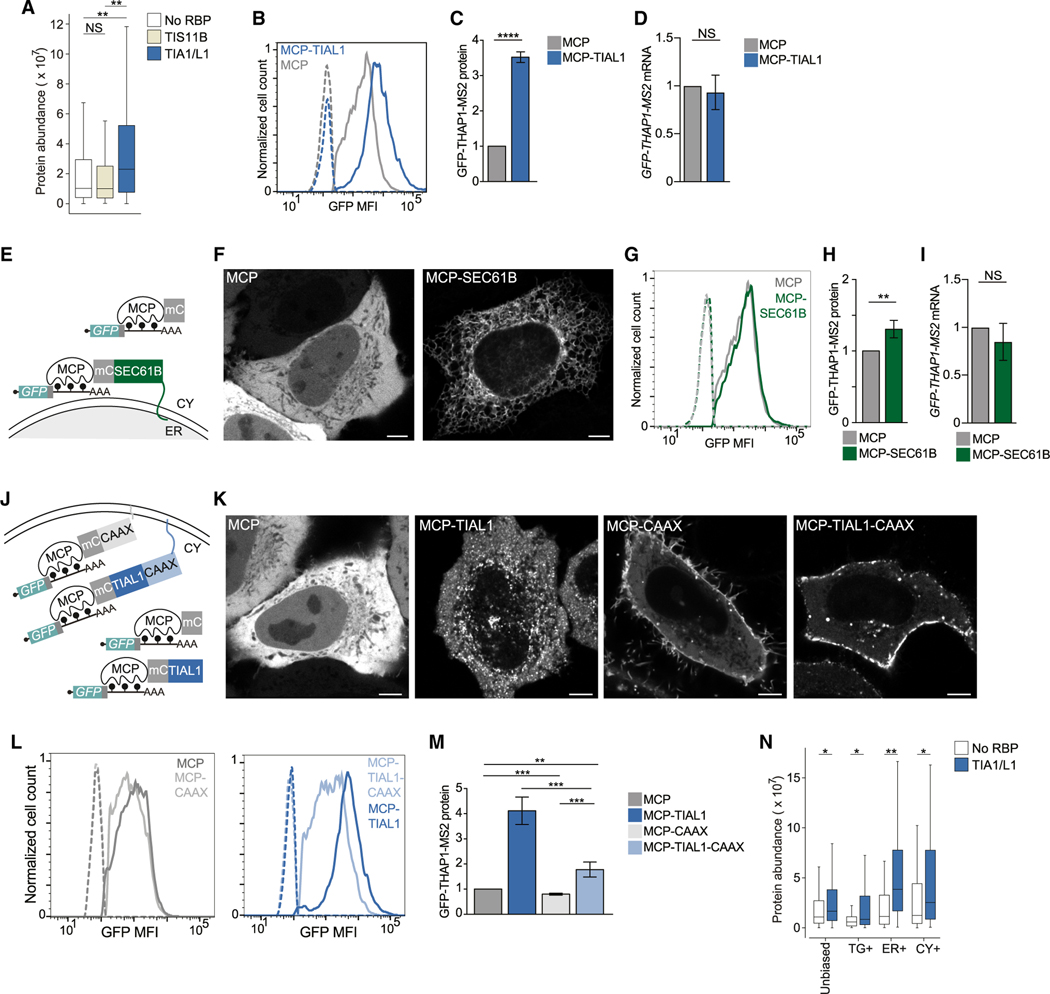
3′ UTR-bound TIAL1 cooperates with the rough ER membrane environment to increase protein expression (A) Protein abundance of mRNAs stratified by RBP binding. No RBP (N = 126), bound by TIS11B (N = 267), bound by TIA1/L1 (N = 232). Mann-Whitney test p values as in Figure 2A. Boxplots depict median, 25th and 75th percentiles (box), and 5% and 95% confidence intervals (error bars). (B) GFP protein expression in HeLa cells using the GFP-THAP1 reporter mRNA with and without TIAL1 tethering. Representative histograms are shown. Dotted lines: GFP-negative cells. (C) Quantification of (B) as mean ± SD of five independent experiments. t test for independent samples, ****p = 0.0003. (D) Quantification of mRNA level from (B) as mean ± SD of three independent experiments. t test for independent samples. (E) Schematic of GFP-THAP1 mRNA reporter to investigate the influence of subcellular mRNA localization on protein expression. MCP-SEC61B fusion localizes the reporter mRNA (as in Figure 4F) to the rough ER membrane, MCP localizes it to the cytosol. (F) Confocal live cell imaging of HeLa cells. Scale bars, 5 μm. (G) As in (B), but reporter mRNA was used with and without SEC61B tethering. (H) Quantification of (G) as mean ± SD of four independent experiments. t test for independent samples, **p = 0.0026. (I) Quantification of mRNA level from (G) as mean ± SD of three independent experiments. t test for independent samples, NS, not significant. (J) As in Figure 4F. Addition of prenylation signal (CAAX) localizes the TIAL1-bound reporter mRNA to the plasma membrane. In the absence of CAAX, the TIAL1-bound reporter mRNA localizes to the rough ER. (K) Confocal live cell imaging of HeLa cells. Scale bars, 5 μm. (L) As in (B) but the reporter mRNA was tethered with the indicated constructs. (M) Quantification of (L) as mean ± SD of four independent experiments. t test for independent samples, ****p < 0.0006, **p = 0.002. (N) Endogenous mRNAs bound by TIA1/L1 encode higher expressed proteins than mRNAs not bound by any RBP. The largest TIA1/L1-associated increase was observed for ER+ mRNAs. Mann-Whitney test p values as in Figure 2A.

For endogenous mRNAs, ER+ mRNAs encode the highest expressed proteins (Figure 2B). Moreover, TIA1/L1-bound mRNAs encode proteins with higher expression levels than other mRNAs (Figure 5A). Using our mRNA reporter (Figure 4F), we investigated the contribution of TIAL1 to steady-state protein expression. We used fluorescence-activated cell sorting (FACS) to measure GFP protein expression of the mRNA reporter, with or without tethering of TIAL1 (Figures S6A–S6C). We observed a 3.5-fold increase in protein expression upon 3′ UTR-tethering of TIAL1 compared with tethering of MCP alone (Figures 5B and 5C). Higher GFP protein expression was not caused by increased mRNA abundance (Figure 5D). We confirmed TIA1/ L1-dependent protein upregulation with a second GFP reporter (Figures S6D–S6F). As TIAL1 promotes translation of mRNAs on the ER membrane, it was unclear whether increased protein expression was caused by TIAL1 or by a potentially unique translation environment provided by the rough ER membrane. For example, mRNAs that encode non-membrane proteins contain 1.4-fold more ribosomes when translated on the ER membrane than when translated in the cytosol.^40^

### TIAL1 cooperates with the rough ER environment to promote protein expression

To disentangle the effects of TIAL1 and the ER membrane on protein expression, we tethered the reporter mRNA directly to the ER surface by fusing MCP to SEC61B, a subunit of the translocon complex in the rough ER (Figure 5E). This recruited the reporter mRNAs to the ER, but only increased protein expression by 1.25-fold compared with the tethering of MCP alone (Figures 5F–5H and S6G–S6I). This approach did not increase mRNA abundance of the reporter (Figure 5I). We used a second ER localization reporter by fusing MCP to TRAPα, a different subunit of the translocon complex, and observed a 1.5-fold increase in protein expression (Figures S6J–S6M). These results suggested that the ER membrane environment has a significant but small stimulatory effect on translation.

Next, we investigated whether the TIAL1-dependent increase in protein expression is intrinsic to TIAL1 or whether it depends on its localization to the ER membrane. We added a CAAX motif to TIAL1 to localize the TIAL1-bound mRNA reporter to the plasma membrane instead of the ER membrane (Figure 5J). The CAAX signal is a prenylation motif that efficiently localized MCP and MCP-TIAL1 to the plasma membrane (Figure 5K).^32^ Translation of the TIAL1-bound mRNA reporter at the plasma membrane increased protein expression by 1.8-fold (Figures 5L and 5M). As translation of the TIAL1-bound reporter at the ER membrane resulted in 2-fold higher protein expression than its translation at the plasma membrane (Figure 5M), our result suggested that TIAL1 cooperated with the environment on the rough ER membrane to promote protein expression.

As the RBPs bound to the reporter mRNA were identical in these experiments, our results demonstrate that the subcytoplasmic location of translation controls steady-state protein expression levels by 2-fold when comparing plasma and ER membranes. This relationship was also observed for endogenous mRNAs, where TIA1/L1-bound mRNAs were associated with high protein output in every compartment, but with the highest protein yields being observed in the ER compartment (Figure 5N).

### The repressive effect of cytosolic TIS11B on protein expression is overcome by its localization to the rough ER membrane

**Figure 6.**
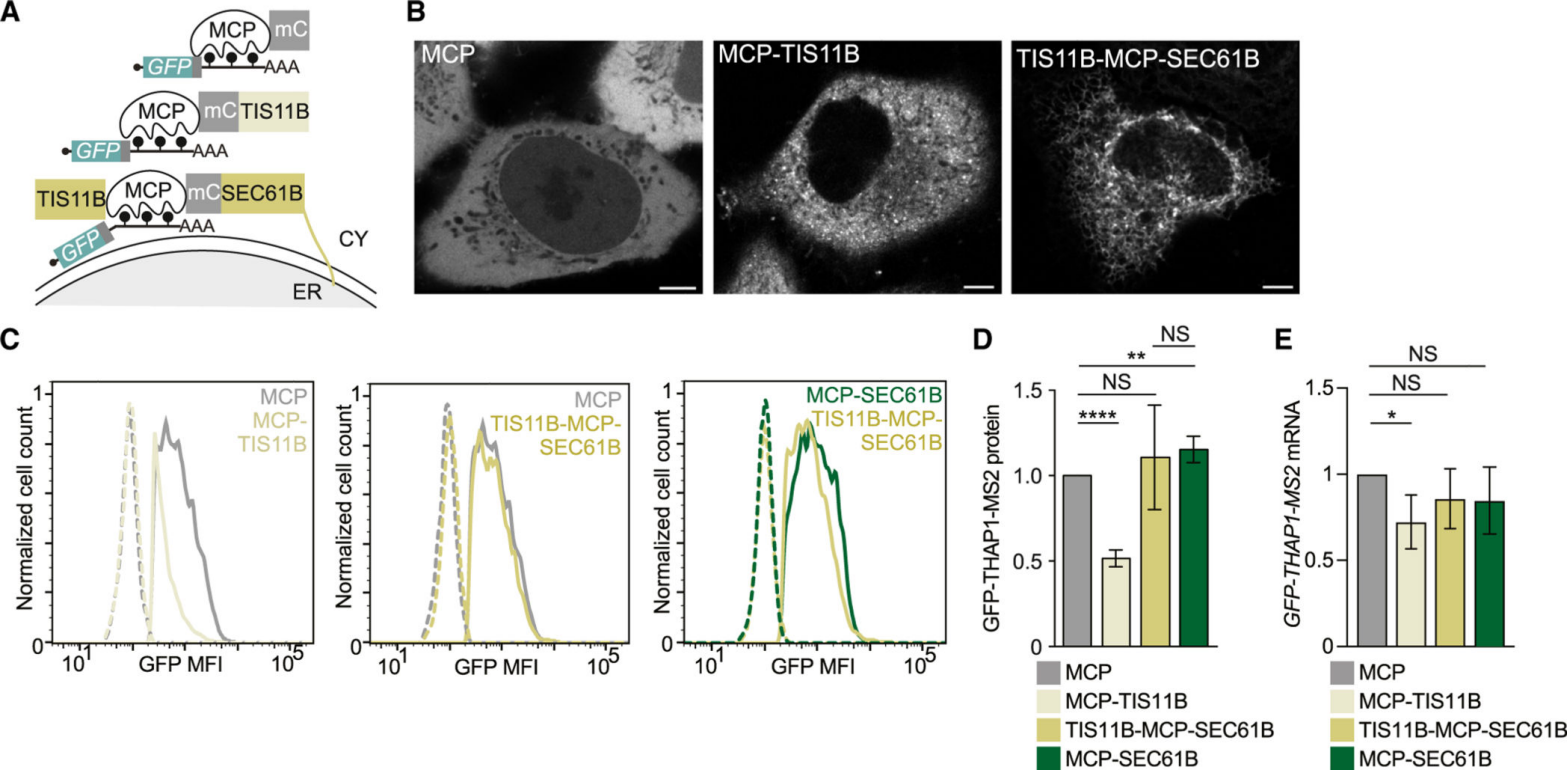
Localization of cytosolic mRNAs to the rough ER membrane increases their protein expression (A) Schematic of a GFP-THAP1 reporter mRNA bound by TIS11B to investigate localization-dependent GFP protein expression. MCP-TIS11B fusion localizes the mRNA reporter to the cytosol. TIS11B-MCP-SEC61B fusion localizes the mRNA reporter to the rough ER membrane. (B) Confocal live cell imaging of HeLa cells expressing constructs from (A). Scale bars, 5 μm. (C) As in Figure 5B. (D) Quantification of (C) as mean ± SD of four independent experiments. t test for independent samples, ****p < 0.0001, **p = 0.003. (E) Quantification of mRNA level in the experiment from (C). Shown is the mean ± SD of three independent experiments. t test for independent samples, *p = 0.037; NS, not significant.

Next, we examined whether the ER environment also promotes protein expression of mRNAs bound by other RBPs, including TIS11B (Figures 6A and 6B). In cells expressing mCherry-TIS11B fusion constructs, about 30% form TGs at steady state (Figures S7A and S7B).^16^ However, addition of MCP to TIS11B fusion constructs resulted in limited TG formation and predominant expression of TIS11B in the cytosol (Figures S7A and S7B). In the cytosolic state, binding of MCP-TIS11B to the reporter mRNA repressed reporter protein expression by 2-fold, compared with tethering of MCP alone (Figures 6C and 6D). This decrease in protein expression was partially caused by a TIS11B-dependent decrease in mRNA level (Figure 6E), consistent with previous reports that suggested that cytosolic TIS11B represses the expression of certain cytokine and cell cycle mRNAs.^41–43^ In contrast, fusing TIS11B to MCP-SEC61B localizes TIS11B and the bound reporter mRNA to the rough ER (Figures 6A and 6B), which overcomes the repressive effect of cytosolic TIS11B and increases protein expression 2-fold (Figures 6A–6E). The 2-fold increase in protein expression was recapitulated with a second reporter and indicates that the repressive effect on protein expression mediated by cytosolic TIS11B is overcome by translation of the TIS11B-bound mRNA on the ER (Figures 6D and S7C–S7E).

### Model

**Figure 7.**
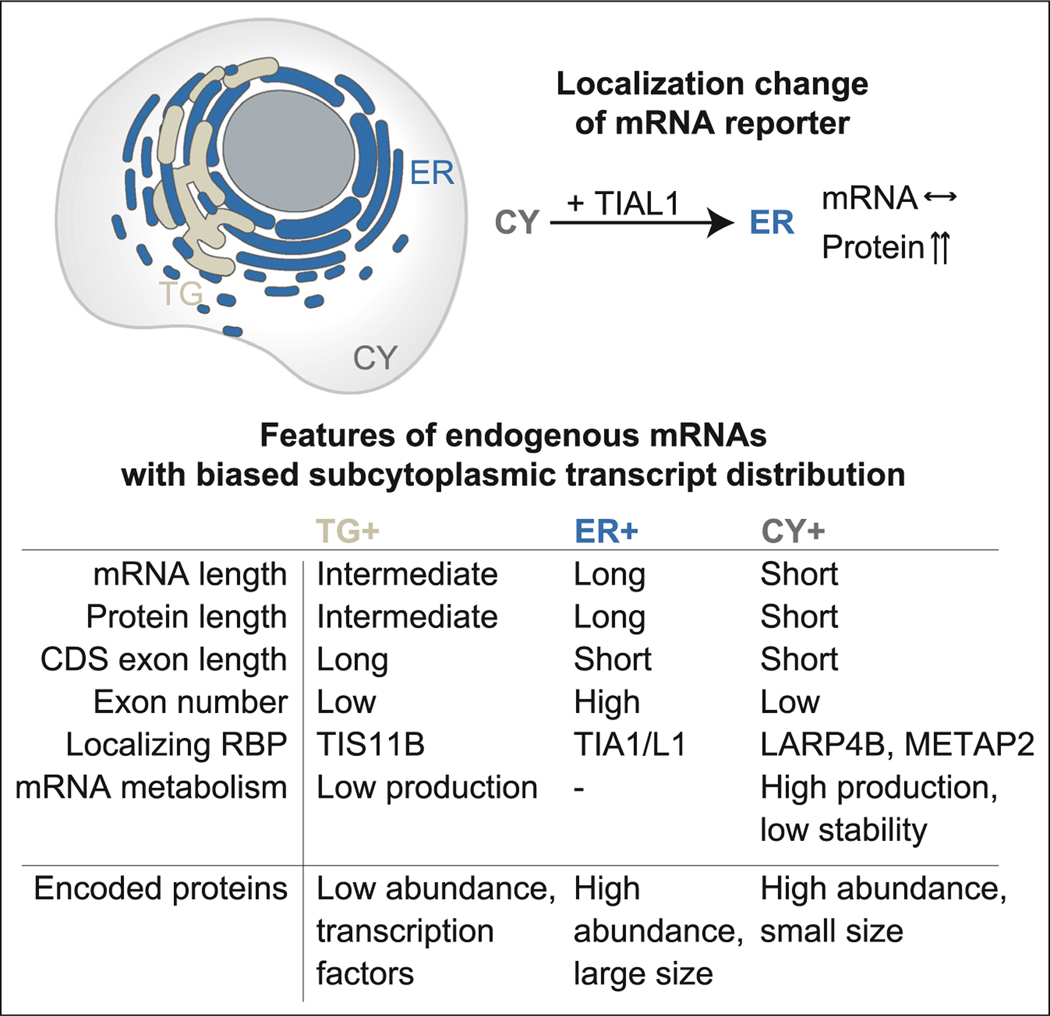
Model Model showing features of endogenous mRNAs with biased subcytoplasmic transcript distribution. See text for details. Horizontal arrow: no change.

Taken together, we observed that mRNAs that are uniquely enriched in one of three cytoplasmic compartments differ substantially in their architectural features, in the RBPs bound to them, and in the expression levels and functional classes of their encoded proteins (Figure 7). TG+ mRNAs are characterized by the longest CDS exons and TIS11B binding to the 3′ UTR. These mRNAs encode the lowest abundance proteins with strong enrichment of transcription factors. In contrast, although TGs are intertwined with the rough ER, ER+ mRNAs are the longest, are predominantly bound by TIA1/L1, and encode highly abundant large proteins. CY+ mRNAs are the shortest and encode small and highly abundant proteins. They are bound by LARP4B/METAP2 and have high production and degradation rates. Through mRNA reporters, we showed that relocation of protein synthesis from the cytosol to the ER increases protein expression, indicating that the location of translation influences protein output (Figure 7).

## DISCUSSION

We determined the distribution of endogenous mRNA transcripts across three cytoplasmic compartments, including TGs, the rough ER, and the cytosol under steady-state conditions. Our RNA-seq results, which were validated by smRNA-FISH, suggest that approximately half of the genes that encode non-membrane proteins have transcripts that are uniquely enriched in one of these three cytoplasmic compartments.

### Functionally related genes are translated in unique compartments

One of our most striking findings was that within each investigated compartment a different group of functionally related mRNAs is translated (Figure 2). Moreover, the compartment-enriched mRNAs have vastly different gene architectures and are characterized by substantially different production and degradation rates as well as the expression levels of their encoded proteins (Figure 2). These features are consistent with the compartment-enriched gene groups, indicating that the cytoplasm is strongly partitioned into different functional and regulatory compartments that are not enclosed by membranes.

Surprisingly, we observed that transcription factors are strongly enriched among TG+ mRNAs (Figure 2J). This unexpected result can be explained by the previous observation that transcription factors are often present at low abundance,^31^ and we found that TG+ mRNAs encode the proteins with the lowest expression levels (Figure 2B). Moreover, many transcription factors have an unusual gene architecture with longer than average coding exons. Together with TIS11B binding, this feature correlated the strongest with mRNA enrichment in TGs (Figures 3C–3E). Interestingly, both characteristics are associated with features associated with low mRNA abundance levels, but whereas TIS11B-binding correlates negatively with pre-mRNA production rates (Spearman’s correlation coefficient R = −0.26), CDS exon length negatively correlates with mRNA half-life (Spearman’s correlation coefficient R = −0.34).^44^ The unique gene architecture together with predominant binding of TIS11B provides an explanation for why TGs enrich for low-abundance mRNAs.

In contrast, ER+ mRNAs encode the largest proteins with the highest expression levels. These include helicases, cytoskeleton-bound proteins, and chromatin regulators (Figure 2). It is possible that anchoring of ribosomes on the ER membrane may facilitate the protein synthesis of very large proteins. Moreover, it is notable that, despite the intertwinement of TGs and the rough ER, the compartment-enriched mRNAs encode proteins that differ substantially in their expression levels and which are the lowest for TG+ mRNAs and the highest for ER+ mRNAs.

It was previously shown that localization to the ER membrane of certain non-membrane protein encoding mRNAs increases their translation,^9,40^ and we confirmed this result. In addition, we describe a so far undescribed role for TIAL1 in the regulation of translation, as TIAL1 binding substantially increased mRNA translation (Figure 5C). So far, TIA1 and TIAL1 have mostly been described as regulators of pre-mRNA splicing and as translational repressors in the context of cellular stress, where they assemble into stress granules.^45,46^ However, in the absence of stress, TIA1/L1 has been reported to promote polysome association, which supports our findings.^36,47^ For both reporter mRNAs and endogenous mRNAs, we observed that the presence of TIAL1 increased protein expression in all compartments, but only in the context of the ER did we observe a cooperative effect on translation (Figures 5M and 5N). The factor that cooperates with TIAL1 on the ER to upregulate translation is currently unknown. Importantly, our reporter results demonstrate that a change in the location of protein synthesis within the cytoplasm strongly influences protein output, indicating that a change in mRNA localization can alter protein abundance.

### Subcytoplasmic mRNA transcript distribution correlates with a combinatorial code of mRNA architecture features and 3′ UTR-bound RBPs

RBPs play an established role in mRNA localization.^1,7^ Additionally, we observed a strong association of mRNA architecture features with transcript localization to the three compartments (Figure 3C). It is possible that mRNA length, CDS length, and CDS exon length do not directly regulate mRNA localization but that specific factors read-out the information. We speculate that mRNA architecture influences messenger ribonucleoprotein (mRNP) size, conformation, and packaging^48,49^ and that these biophysical features act as additional determinants of subcytoplasmic mRNA localization. This idea is supported by previous insights into *oskar* mRNA localization, where the deposition of the exon junction complex, involved in mRNP packaging,^48,49^ was required for proper mRNA localization in the cytoplasm.^50^

We present a model for the regulation of subcytoplasmic transcript distribution that is based on a combinatorial code generated by mRNA architecture features together with the bound RBPs, where individual components act in an additive manner (Figures 3E and 3G). This model was tested experimentally by analyzing the localization propensity of TG+ mRNAs upon deletion of TIS11B. This experiment confirmed the contribution of mRNA architecture features to mRNA localization and suggests that the binding of RBPs overcomes the default localization pattern established by gene-intrinsic features (Figures 4A–4E).

### Is it biologically relevant if only 20% of transcripts localize to TGs?

Based on the estimated size of TGs (Figure 1I), we expect that 11% of mRNA transcripts localize to TGs by chance. Using smRNA-FISH on TG+ mRNAs, we observed a 2-fold enrichment in TGs, meaning that, on average, 22% of these transcripts localize to TGs. This raises the important question of whether it matters biologically if a minority population of transcripts for a given mRNA localizes to a certain compartment.

This question was addressed in a follow-up project, where we investigated the biological consequences of *MYC* mRNA, which is a TG+ mRNA, when it was translated in TGs or the cytosol.^19^ We observed that several MYC protein complexes were only formed when *MYC* mRNA was translated in TGs and not when it was translated in the cytosol. The TG-dependent protein complexes formed co-translationally and had functional consequences for MYC target gene expression in the nucleus. TG-translated MYC induced different target genes than cytosol-translated MYC.^19^ Our results indicate biological relevance, even when only a fraction of transcripts are translated in TGs.

In summary, our study revealed a surprisingly high degree of cytoplasmic compartmentalization. This is the basis for the translation of functionally related proteins in defined environments that strongly affect mRNA and protein expression. Our results highlight the contribution of spatial regulation, whose consequences go beyond the effects mediated by the mRNA-bound proteins. In the future, our findings may provide the basis for biotechnology applications that make use of engineered 3′ UTR sequences to boost protein expression in experimental settings or to increase protein production of mRNA vaccines.

### Limitations of the study

The exact compartment sizes of TGs, the rough ER, and the cytosol are currently unknown and can only be estimated. However, compartment-enriched mRNAs were identified using two different methods, which yielded highly similar results.

To obtain sufficient material for TG and ER particle sorting, we used transfected, fluorescently labeled proteins instead of endogenous proteins. We did not knock down TIS11B or SEC61B, as overexpression had minimal effects on the transcriptome, while the knockdown of TIS11B changed the abundance of thousands of transcripts. In the future, TG particle sorting may be possible using endogenous, fluorescently tagged TIS11B in cells with high TIS11B expression.

The use of spike-ins to isolated compartments obtained from defined cell numbers may have enabled us to perform absolute, versus the relative, enrichment analyses that we report here. Moreover, all analyses were performed at the gene level. Alternative 3′ UTR isoforms are known to differentially localize and, therefore, we would expect to obtain a higher resolution for compartment enrichment of transcripts if, instead of genes, alternative 3′ UTR isoforms had been analyzed.^38,51^ However, with our purification strategy we did not obtain sufficient mRNA quantities to perform the study at the level of alternative 3′ UTRs.

## STAR★METHODS

### RESOURCE AVAILABILITY

#### Lead contact

Further information and requests for resources and reagents should be directed to and will be fulfilled by the lead contact, Christine Mayr (mayrc@mskcc.org).

#### Materials availability

- Plasmids generated in this study have been deposited to Addgene.
- Plasmids generated in this study not available at Addgene are available from the lead contact.
- The TIS11B inducible knockout HEK293T cell line (together with the control cell line) generated in this study are available from the lead contact with a completed Materials Transfer Agreement.

#### Data and code availability

- The data of the proteomics experiment were deposited in the MassIVE repository (dataset identifier: MSV000092176). The RNA-seq samples obtained from the subcytoplasmic fractionation and the TIS11B iCLIP data obtained from HEK293T cells are available at GEO (Accession number: GSE215770). The code for logistic regression is available on Zenodo (https://doi.org/10.5281/zenodo.10056230). Western blot data, raw imaging data and scripts for analysis are deposited at Mendeley (https://data.mendeley.com/datasets/nmt7ppsp8r/1).
- All original code has been deposited at Zenodo and is publicly available as of the date of publication. DOIs are listed in the key resources table.
- Any additional information required to reanalyze the data reported in this paper is available from the lead contact upon request.

### EXPERIMENTAL MODEL AND STUDY PARTICIPANT DETAILS

#### Cell lines

HEK293T (human immortalized embryonic kidney cells, female origin) was purchased from ATCC. HeLa, a human cervical cancer cell line (female origin), was a gift from the lab of Jonathan S. Weissman (UCSF), provided by Calvin H. Jan. All cells were maintained at 37°C with 5% CO2 injection in Dulbecco’s Modified Eagle Medium (DMEM) containing 4,500 mg/L glucose, 10% heat inactivated fetal bovine serum, 100 U/ml penicillin and 100 μg/ml streptomycin.

#### Generation of a doxycycline inducible *TIS11B* knockout cell line (*TIS11B* KO)

Doxycycline inducible Cas9 (iCas9) HEK293T cells were generated by infecting cells with lentivirus containing a Cas9-P2A-GFP expression cassette under a doxycycline inducible promoter as described previously.^53^ During consecutive rounds of fluorescence-activated cell sorting, we selected a cell pool exhibiting robust induction of Cas9/GFP expression after doxycycline treatment (100 ng/ml for 24 hours), and low levels of leaky transgene expression in the absence of the drug. Next, we transduced iCas9 cell lines with a lentiviral construct harboring a pair of guide RNAs either targeting *TIS11B* or gRNAs that target an intergenic region. To generate these constructs, we adapted the plentiGuide-puro vector.^54^ to incorporate a second guide RNA expression cassette as described previously.^55^ For this purpose, the plasmid was digested with BsmBI (FastDigest Esp3I, Thermo Fisher Scientific) and a synthetic 391 bp double-stranded DNA fragment encoding 5′-(1st gRNA/scaffold/H1 promoter/2nd gRNA)-3′ was inserted using the NEBuilder HiFi assembly system (NEB). Synthetic DNA fragments were ordered from Genewiz and sequences are listed in Table S6. The assembled vector DNA was used to transform chemically competent Stbl3 bacteria cells (Invitrogen), and correct vector clones were identified by Sanger sequencing.

Lentivirus was generated in HEK293T cells using standard methods and 200 μl of viral supernatant was used to transduce iCas9 cells in a 6-well dish together with 8 μg/ml polybrene. Transduced cells were subjected to puromycin selection (1 μg/ml) for five days and resistant cells were aliquoted and frozen for all further experiments. Finally, for induction of gene knockouts, *TIS11B* KO and corresponding control cells (with gRNAs targeting an intergenic region) were treated with doxycycline (100 ng/ml) for five days, after which TIS11B protein expression was evaluated by western blotting, ER particle sorting and digitonin extraction was performed.

### METHOD DETAILS

#### Constructs

##### Fluorescently-tagged TIS11B and SEC61B constructs

The eGFP/mCherry/BFP fusion constructs for TIS11B and SEC61B expression were described previously.^16^ They were generated in the pcDNA3.1-puro expression vector. The TIS11B and SEC61B coding regions were PCR amplified from HeLa cDNA and inserted downstream of eGFP/mCherry/BFP using BsrGI/EcoRI or BsrGI/HindIII restriction sites, respectively.

##### Constructs to generate the mRNA localization reporter

To investigate the influence of RBPs on mRNA localization of a GFP mRNA reporter, RBPs were fused to MCP and tethered to a GFP mRNA reporter containing MS2 binding sites as 3′UTR.^37,38^ To investigate mRNA localization-dependent protein expression of the GFP mRNA reporter, a CAAX sequence was fused to MCP or to MCP-RBP fusions.

##### GFP mRNA reporter

To generate the GFP mRNA reporter, the GFP-BIRC3-MS2-SU^39^ vector was used the BIRC3 coding region was replaced with the THAP1 coding region. It was PCR amplified from the GFP-THAP1 vector using THAP1-MS2 F and THAP1-MS2 R primers and inserted between the BsrGI and AgeI sites. The SU fragment was removed with HindIII and XhoI and blunt end ligated, resulting in GFP-THAP1-MS2.

##### MCP-mCherry RBP fusion constructs

To generate MCP-mCherry, the MCP coding sequence was PCR amplified from UBC NLS-HA-2XMCP-tagRFPt vector (Addgene 64541) using MCP F and MCP R primers and inserted in-frame, upstream of mCherry (mCherry lacking a start codon) between BmtI and BamHI sites in pcDNA3.1-puro-mCherry vector.^16,52^ To generate MCP-mCherry-TIS11B and MCP-mCherry-TIAL1, their coding sequences were inserted in-frame, downstream of mCherry between the BsrGI and XbaI sites. The TIS11B coding sequence was amplified from pcDNA3.1-puro-GFP-TIS11B using TIS11B MCP F and TIS11B MCP R primers and the TIAL1 coding sequence was PCR amplified from pFRT_TO_FlagHA_TIAL1 (Addgene 106090) using TIAL1 MCP F and TIAL1 MCP R primers.

##### MCP-mCherry fusion constructs with subcellular localization signals

To generate pcDNA3.1-puro-MCP-mCherry-SEC61B, the MCP-mCherry coding sequence was cut from MCP-mCherry vector using BmtI and BsrGI and pasted in-frame, upstream of SEC61B in pcDNA3.1-mCherry-SEC61B (replacing mCherry). To generate the TIS11B-MCP-mCherry-SEC61B vector, TIS11B coding sequence was PCR amplified from pcDNA3.1-puro-GFP-TIS11B using TIS-SEC F and TIS-SEC R primers and pasted in-frame, upstream of MCP into the BmtI site in the MCP-mCherry-SEC61B vector. To generate TRAPα-MCP-mCherry, the TRAPα coding sequence (encoded by the *SSR1* gene) was PCR amplified from HeLa cDNA using TRAPα MCP F and TRAPα MCP R and inserted in-frame, upstream of MCP in the pcDNA3.1-puro-MCP-mCherry vector.

For plasma membrane localization, the CAAX prenylation signal was added to the C-terminus of MCP-mCherry or MCP-mCherry-TIAL1. The CAAX coding sequence was purchased as a gene fragment from Azenta as described^32^ and PCR amplified using TIAL1 CAAX F and CAAX R primers. It was inserted in-frame using the BsrGI and ApaI sites, located downstream of mCherry to generate pcDNA3.1-puro-MCP-mCherry-CAAX. It was inserted in-frame using EcoNI and ApaI sites to generate MCP-mCherry-TIAL1-CAAX.

SunTag constructs were described previously.^32^

#### Isolation of subcytoplasmic compartments

##### Transfection

HEK293T cells were seeded in six 10 cm dishes (particle sorting) or one well from a 6-well plate (cytosol extraction) at 80% confluency in antibiotic free media. After 24 hours, cells were transfected by calcium phosphate with either 3 μg mCherry-TIS11B or 1 μg GFP-SEC61B per dish (particle sorting), or 500ng mCherry-TIS11B (cytosol extraction). We modestly overexpress mCherry-TIS11B compared to its endogenous levels (Figure S1C), which results in approximately 30% of cells to form TGs. This amount was chosen, because 25–30% of HEK293T cells form TG from endogenous TIS11B.

##### Particle purification

20 hours after transfection, cells were rinsed once with ice-cold PBS, scraped in 10 ml ice-cold PBS, and pelleted at 300 × g. Pellets from two plates were resuspended in 1 ml ice-cold hypotonic isolation buffer (225 mM mannitol, 75 mM sucrose, 20 mM Tris-HCl pH 7.4, 0.1 mM EDTA). Cells were lysed with 50 strokes in a 1 ml dounce-homogenizer with pestle on ice in order to shear the nuclei from the ER. Nuclei were pelleted with a two-minute spin at 600 × g. The supernatant contains the cytoplasmic membrane fraction, which was pelleted with a 15-minute spin at 7000 × g and resuspended in ice-cold PBS for fluorescent particle sorting.

##### Fluorescent particle sorting

Particles were sorted on a BD FACSAria III cell sorter equipped with a 70 μm nozzle. The forward-scatter threshold was decreased from 5,000 to 800 in order to visualize subcellular particles. Particles were first detected by fluorescence using the 594 nm and 488 nm excitation lasers, for mCherry-TIS11B and GFP-SEC61B respectively, and 405 nm excitation laser for DAPI. A sorting gate was drawn on particles that were either mCherry-positive or GFP-positive, but DAPI-negative, to exclude any remaining nuclei. Sorting was performed in purity mode with an average speed of 150 particles/second. Particles were sorted directly into 1 ml of TRIzol solution in Eppendorf tubes, holding 180,000 particles per tube. RNA extraction was performed for each tube separately and total RNA for each sample was combined for library preparation. Two biological replicates for each particle prep were sequenced. For each replicate, about 1.5 million TIS11B granule particles and 2.0 million ER particles were collected.

##### Cytosol extraction

The cytosol was extracted as previously described.^24^ HEK293T cells transfected were plated in a six-well plate at 80% confluency. After 24 hours, cells were rinsed once in the dish with ice-cold PBS. After aspirating PBS, 300 μl ice-cold digitonin solution (40 μg/ml digitonin, 150 mM NaCl, 20 mM HEPES pH 7.4, 0.2 mM EDTA, 2 mM DTT, 2 mM MgCl2) was added and incubated on a shaker at 4°C for ten minutes. After incubation, the digitonin-derived cytosolic extract was pipetted from the plate and spun at 20,000 × g for one minute to pellet any floating cells. 200 μl of cytosolic extract was added to 1 ml Trizol solution for RNA extraction.

##### RNA-seq library preparation

RiboGreen RNA Reagent (ThermoFisher) was used for RNA quantification and quality control was performed by Agilent BioAnalyzer. 50–500 ng of total RNA underwent polyA selection and TruSeq library preparation according to instructions provided by Illumina (TruSeq Stranded mRNA LT Kit, catalog # RS-122–2102), with eight cycles of PCR. Samples were barcoded and run on a HiSeq 4000 in a PE50 run, using the HiSeq 3000/4000 SBS Kit (Illumina). An average of 27 million paired reads was generated per sample.

#### Western Blotting

For whole cell lysate preparation, cells were trypsinized and washed twice with PBS and lysed in 2x Laemmli Sample buffer (Alfa Aesar, J61337). For cytosolic lysate, cytosol was extracted with digitonin as described above and one volume of 2x Laemmli Sample buffer was added. Laemmli lysates were boiled for 10 min at 95°C. Samples were subjected to SDS-PAGE on NuPAGE 4%–12% Bis-Tris gradient protein gel (Invitrogen). Imaging was captured on the Odyssey DLx imaging system (Li-Cor). Quantification was performed using ImageJ. The antibodies used are listed in the key resources table.

#### TIS11B iCLIP

##### Transfection

HEK293T cells were seeded in 10 cm dishes at 80% confluency in antibiotic free media. After 24 hours, cells were transfected by calcium phosphate with either 3 μg GFP-TIS11B or 1.5 μg GFP-only per dish.

##### Sample preparation

20 hours after transfection, cells were rinsed once with ice-cold PBS and 6 ml of fresh PBS was added to each plate before cross-linking. Cells were irradiated once with 150 mJ/cm^2^ in a Spectroline UV Crosslinker at 254 nm. Irradiated cells were scraped into Eppendorf tubes, spun at 500 × g for one minute, and snap-frozen. Crosslinked cell pellets were lysed in iCLIP lysis buffer (50 mM Tris-HCl pH 7.4, 100 mM NaCl, 1% Igepal CA-630 (Sigma I8896), 0.1% SDS, 0.5% sodium deoxycholate), sonicated with the Bioruptor Pico for 10 cycles 30 seconds ON/30 seconds OFF, and supplemented with 0.5 U of RNase I per 1 mg/ml lysate for RNA fragmentation. Lysates were pre-cleared by centrifugation at 20,000 × g at 4°C. A mix of Protein A/G Dynabeads (50 μl of each per sample, Life Technologies) were coupled to 10 μg of rabbit anti-GFP antibody (Abcam ab290). TIS11B protein-RNA complexes were immunoprecipitated from 1 ml of crosslinked lysate and washed with high salt and PNK buffer (NEB). RNA was repaired by 3′ dephosphorylation and ligated to L3-IR adaptor on beads.^56^ Excess adaptor was removed by incubation with 5′ deadenylase and the exonuclease RecJf (NEB). TIS11B protein-RNA complexes were eluted from the beads by heating at 70°C for one minute. The complexes were then visualized via the infrared-labeled adaptor, purified with SDS-PAGE, and transferred to nitrocellulose membrane. cDNA was synthesized with Superscript IV Reverse Transcriptase (Life Technologies) and circularized by CircLigase II. Circularized cDNA was purified with AMPure bead-based purification (A63880, Beckman Coulter), amplified by PCR and sequenced by Novaseq.

#### RNA-FISH

##### Single molecule RNA-FISH for endogenous mRNAs

###### Probe design.

Primary probes were designed using the ProbeDealer package in MATLAB.^57^ Each primary probe contains 30 transcript-targeting nucleotides preceded by 20 common nucleotides that are complementary to the secondary probe. At least 30 probes were designed for each transcript, purchased in a pool from IDT. The secondary probes are 5′ conjugated to AlexaFluor 633 and were purchased from IDT.

##### Transfection

Prior to cell seeding, 35 mm glass cover slips were sterilized with ethanol then incubated in 1 μg/ml fibronectin in PBS at room temperature for one hour. Cover slips were rinsed in PBS and HeLa cells were seeded at 100,000 per coverslip. 24 hours after seeding, cells were co-transfected with 250 ng BFP-TIS11B and 100ng of GFP-SEC61B using Lipofectamine 3000 (Invitrogen).

##### Sample preparation

20 hours after transfection, cells were rinsed once with PBS then fixed in 4% paraformaldehyde for 10 minutes at room temperature. All steps were performed at room temperature if not otherwise noted. Cells were rinsed twice with PBS and permeabilized with 0.5% Triton-X solution for 10 minutes. Cells were rinsed twice with PBS and incubated for five minutes in pre-hybridization buffer (2xSSC, 50% formamide). Cells were incubated in primary probe hybridization solution (40 μM primary probe, 2xSSC, 50% formamide, 10% dextran sulfate (Sigma), 200 μg/ml yeast tRNA (Sigma), 1:100 Murine RNase Inhibitor (NEB)), for at least 15 hours at 37°C. To remove excess or unbound primary probes, cells were then rinsed twice in 2xSSC + 0.1% Tween for 15 minutes at 60°C then once more for 15 minutes at room temperature. Cells were incubated in secondary probe solution (4 nM secondary probe, 2xSSC, 50% ethylene carbonate, 1:100 Murine RNase Inhibitor) for 30 minutes in the dark. Secondary probes were rinsed twice in 50% ethylene carbonate, 2xSSC solution for five minutes then mounted with Prolong Diamond mounting solution (Invitrogen).

##### Cytosol extraction

To visualize and validate CY+ versus TG+ or ER+ endogenous mRNAs, HeLa cells were seeded as described above, then incubated in 2 ml digitonin solution described above (40 μg/ml digitonin, 150 mM NaCl, 20 mM HEPES pH 7.4, 0.2 mM EDTA, 2 mM DTT, 2 mM MgCl2) for 10 min at 4°C. Digitonin solution was removed, coverslips were rinsed with 2 ml PBS, and RNA-FISH was performed as described above. Mounting media with DAPI was used to visualize nuclei (Invitrogen P36931).

##### Validation of TG+ and ER+ mRNAs using smRNA-FISH

We performed smRNA-FISH on endogenous mRNAs (Table S2) while simultaneously visualizing TGs and the ER. We considered an mRNA to have an unbiased localization pattern if its transcript distribution correlated with the cytoplasmic compartment sizes. As a proxy for the relative compartment sizes, we used the area occupied by TGs or the ER compared to the whole cell area, obtained from the maximum projection of the fluorescent signals in 186 cells. We used FIJI to delineate the whole cell border with the fluorescent signal from RNA-FISH. For TGs, the fluorescent signal from BFP-TIS11B and for the ER the fluorescent signal from GFP-SEC61B both obtained from the maximum intensity Z-projections was used to delineate each compartment. Where there was overlap between the TG mask and the ER mask, the ER was subtracted, and the region was defined as TG. In this way the compartments are mutually exclusive. The mask area of each compartment was quantified and read out as a proportion of the total cell area. Across all cells, the median size of TGs was estimated to be 11% of the cell size, whereas the median ER size was estimated to be 29% of the cell size (Figures 1I and 1J). Therefore, for mRNAs with an unbiased transcript distribution, we expect that typically 11% of transcripts colocalize with TGs and 29% colocalize with the ER.

To determine mRNA transcripts enriched in TG or ER, smRNA-FISH foci were counted using the maxima function and the total number of foci per cell are quantified. Next, all foci are overlaid with the TG mask and the ER mask to identify mRNAs that colocalize with each compartment. To determine if an mRNA is compartment enriched, we tested if its observed compartment distribution differs from the expected distribution based on compartment size using a Mann Whitney test. The code for the image analysis is available (see below).

Of note, this analysis does not distinguish between nuclear and cytoplasmic mRNA localization. For 7/8 mRNAs this does not influence the outcome because the mRNA signal in the nucleus is negligible or non-existent. However, smRNA-FISH probes for endogenous *TES* produce high nuclear background signal. In this case, the prominence value, used to define local maxima to call foci, is increased such that nuclear noise does not substantially influence foci quantification (Figure S2G).

##### Validation of CY+ mRNAs by smRNA-FISH after digitonin extraction

To distinguish CY+ mRNAs from TG+ or ER+ mRNAs, we performed smRNA-FISH on endogenous mRNAs in untreated and digitonin treated cells, as previously reported.^58^ The total number of mRNA foci per cell is calculated using the maxima function in FIJI. Next, thresholding is applied to DAPI fluorescence to generate a nuclear mask. Total mRNA foci are overlaid with the DAPI mask and nuclear foci are subtracted from the total, yielding cytoplasmic foci. Cytoplasmic foci are quantified for at least 10 cells per condition per experiment. For each experiment, the mean fraction of transcripts retained is calculated as the average cytoplasmic foci per digitonin-treated cell divided by the average cytoplasmic foci per untreated cell. At least three separate experiments per mRNA were performed.

##### RNA-FISH after transfection of constructs

RNA-FISH experiments probing for GFP-fusion constructs were performed as described previously.^16^ Stellaris FISH probes for eGFP with Quasar 670 Dye were used.

##### Line profile analysis

To quantify colocalization of ER (GFP-SEC61B) and mRNA (AF633) fluorescence signals, line profiles were generated with FIJI (ImageJ). For each cell, 2–4 straight lines were drawn to cross the ER in different directions, indicated by the white arrows shown in the figures. Fluorescence signal along the straight line of the ER and the mRNA reporter was calculated for each channel using the plot profile tool in FIJI. The values of the Pearson’s correlation coefficient r were calculated using Excel. Perfect correlation of protein-mRNA is indicated by r = 1, perfect exclusion is indicated by r = −1, and random distribution is indicated by r = 0.

#### Confocal microscopy

Confocal imaging was performed using ZEISS LSM 880 with Airyscan super-resolution mode or Nikon CSU-W1 with SoRa super-resolution mode. A Plan-Apochromat 63x/1.4 (Zeiss) or 60x/1.49 (Nikon) Oil objective was used. For live cell imaging, cells were incubated with a LiveCell imaging chamber (Zeiss, Nikon) at 37°C and 5% CO2 and imaged in cell culture media. Excitations were performed sequentially using 405, 488, 594 or 633 nm laser wavelength and imaging conditions were experimentally optimized to minimize bleed-through. Z-stack images were captured with the interval size of 0.2 μm. Images were prepared with FIJI (ImageJ) software.

#### TMT mass spectrometry

To obtain protein expression levels, TMT mass spectrometry analysis was performed on HEK293T cells cultivated in steady-state conditions. Cells were trypsinized and washed three times with ice-cold PBS. Pelleted cells were snap-frozen in liquid nitrogen. Cell pellets were lysed with 200 μl buffer containing 8 M urea and 200 mM EPPS (pH at 8.5) with protease inhibitor (Roche) and phosphatase inhibitor cocktails 2 and 3 (Sigma). Benzonase (Millipore) was added to a concentration of 50 μg/ml and incubated at room temperature for 15 min followed by water bath sonication. Samples were centrifuged at 14,000 g at 4°C for 10 min, and supernatant extracted. The Pierce bicinchoninic acid (BCA) protein concentration assay was used to determine protein concentration. Protein disulfide bonds were reduced with 5 mM tris (2-carboxyethyl) phosphine at room temperature for 15 min, and alkylated with 10 mM iodoacetamide at room temperature for 30 min in the dark. The reaction was quenched with 10 mM dithiothreitol at room temperature for 15 min. Aliquots of 100 μg were taken for each sample and diluted to 100 μl with lysis buffer. Samples were subject to chloroform/methanol precipitation as previously described.^59^ Pellets were reconstituted in 200 mM EPPS buffer and digested with Lys-C (1:50 enzyme-to-protein ratio) and trypsin (1:50 enzyme-to-protein ratio), and digested at 37°C overnight.

Peptides were TMT-labeled as described.^59^ Briefly, peptides were TMT-tagged by the addition of anhydrous ACN and TMTPro reagents (16plex) for each respective sample and incubated for 1 hour at room temperature. A ratio check was performed by taking a 1 μl aliquot from each sample and desalted by StageTip method.^60^ TMT tags were then quenched with hydroxylamine to a final concentration of 0.3% for 15 min at room temperature. Samples were pooled 1:1 based on the ratio check and vacuum-centrifuged to dryness. Dried peptides were reconstituted in 1 ml of 3% ACN/1% TFA, desalted using a 100 mg tC18 SepPak (Waters), and vacuum-centrifuged overnight.

Peptides were centrifuged to dryness and reconstituted in 1 ml of 1% ACN/25mM ABC. Peptides were fractionated into 48 fractions. Briefly, an Ultimate 3000 HPLC (Dionex) coupled to an Ultimate 3000 Fraction Collector using a Waters XBridge BEH130 C18 column (3.5 um 4.6 × 250 mm) was operated at 1 ml/min. Buffer A, B, and C consisted of 100% water, 100% ACN, and 25mM ABC, respectively. The fractionation gradient operated as follows: 1% B to 5% B in 1 min, 5% B to 35% B in 61 min, 35% B to 60% B in 5 min, 60% B to 70% B in 3 min, 70% B to 1% B in 10 min, with 10% C the entire gradient to maintain pH. The 48 fractions were then concatenated to 12 fractions, (i.e. fractions 1, 13, 25, 37 were pooled, followed by fractions 2, 14, 26, 38, etc.) so that every 12^th^ fraction was used to pool. Pooled fractions were vacuum-centrifuged and then reconstituted in 1% ACN/0.1% FA for LC-MS/MS.

Fractions were analyzed by LC-MS/MS using a NanoAcquity (Waters) with a 50 cm (inner diameter 75 μm) EASY-Spray Column (PepMap RSLC, C18, 2 μm, 100 Å) heated to 60°C coupled to an Orbitrap Eclipse Tribrid Mass Spectrometer (Thermo Fisher Scientific). Peptides were separated by direct injection at a flow rate of 300 nl/min using a gradient of 5 to 30% acetonitrile (0.1% FA) in water (0.1% FA) over 3 hours and then to 50% ACN in 30 min and analyzed by SPS-MS3. MS1 scans were acquired over a range of m/z 375–1500, 120K resolution, AGC target (standard), and maximum IT of 50 ms. MS2 scans were acquired on MS1 scans of charge 2–7 using isolation of 0.5 m/z, collision-induced dissociation with activation of 32%, turbo scan, and max IT of 120 ms. MS3 scans were acquired using specific precursor selection (SPS) of 10 isolation notches, m/z range 110–1000, 50K resolution, AGC target (custom, 200%), HCD activation of 65%, max IT of 150 ms, and dynamic exclusion of 60 s.

#### Visualization of translation in TGs

The SunTag system was used to visualize mRNA translation in the cytosol and the TGER domain. Stable expression of td-PP7–3xmCherry (Addgene 74926) and scFv-GCN4-sfGFP (Addgene 60907) was achieved by generating virus in HEK293T cells and transducing HeLa cells. Cells were seeded on 3.5 cm glass bottom dishes (Cellvis, D35–20-1-N). 20 hours later, cells were transfected with either the SunTag vector expressing KIF18B (Addgene 74928) or SunTag-FOS-UTR. At 15 hours post transfection, cells were treated with 100 ng/ml doxycycline for one hour to induce SunTag expression. Confocal imaging was performed as described above. Co-localization of foci was quantified using FIJI.

#### mRNA localization-dependent GFP protein expression

##### Transfection

HeLa cells were seeded in 12-well plates at 80% confluency and transfected with 250 ng GFP-THAP1-MS2 and 250 ng of the MCP-mCherry fusion constructs indicated in the figure (Lipofectamine 3000, Invitrogen). When indicated, GFP-THAP1 or GFP-BIRC3-MS2-SU was used instead of GFP-THAP1-MS2. At 13–15 hours post transfection, cells were analyzed by FACS. For RNA-FISH experiments, cells were seeded at 80% confluency in 4-well slide chambers (Millipore Sigma) and cotransfected with 75 ng GFP-THAP1-MS2, 100 ng BFP-SEC61B, and 75 ng of the indicated MCP-mCherry fusion constructs.

##### FACS analysis to measure GFP protein expression

Cells were trypsinized, washed once in complete media, then resuspended in FACS buffer (PBS plus 1% FCS). At least 5,000 cells were measured on a BD LSR-Fortessa Cell Analyzer and FACS data were analyzed using FlowJo software. GFP protein expression corresponds to GFP mean fluorescence intensity (MFI). To determine the effect of MCP-tethered RBPs on protein output of the GFP reporter mRNA, only cells that were successfully cotransfected with both the MCP-mCherry fusion and the GFP reporter constructs were analyzed. To do so, the double-positive cells (mCherry+/GFP+) were gated, and all single positive and unstained cells were excluded from the analysis. The reported GFP MFI was calculated from the double-positive cells. Untransfected cells were used to draw the gates for mCherry+ or GFP+ cells.

##### *qPCR analysis to measure* GFP *mRNA abundance*

Cells were trypsinized, washed once in complete media, then resuspended in FACS buffer (PBS plus 1% FCS). To determine the effect of MCP-tethered RBPs on GFP reporter mRNA stability, cells were sorted based on expression of both the MCP-mCherry fusion and the GFP reporter constructs. The BD FACSAria III cell sorter was used to collect 50,000 cells from each co-transfected population. Cells were sorted directly into 1 ml of TRIzol solution in Eppendorf tubes for total RNA was extraction. cDNA synthesis was performed on 200 ng of RNA per sample using the SuperScript IV VILO ezDNase Master Mix (Invitrogen). ezDNase enzyme was included to eliminate plasmid DNA contamination. To measure the relative expression levels of reporter mRNA by qRT-PCR, FastStart Universal SYBR Green Master Mix (ROX) from Roche was used together with GFP-qPCR F/R primers. GAPDH was used as a housekeeping gene.

#### Data analysis

##### RNA-seq of subcytoplasmic fractions from HEK293T cells

###### RNA-seq.

Alignment was generated in Dragen v3.10 (Illumina) against the hg38-alt-masked-v2 reference acquired from GENCODE v43 with default parameters. Gene expression analysis was performed using HOMER v4.11 software.^61^ The mean RPKM values of all biological replicates were calculated and used for downstream analyses. Only protein-coding genes were analyzed. A gene was considered expressed if the RPKM value is 3 or greater.

###### Classification of membrane/secretory proteins versus non-membrane proteins.

Information on the presence of transmembrane domains or a signal sequence was obtained from UniProt. All expressed genes were separated into mRNAs that encode membrane/secretory proteins or non-membrane proteins. If a protein contains a signal sequence but not a transmembrane domain, it is considered as secretory protein. All proteins with transmembrane domains are considered membrane proteins and all remaining proteins are classified as non-membrane proteins. Among the 9155 mRNAs expressed in HEK293T cells, 2140 were classified as membrane/secretory proteins, whereas 7015 were classified as non-membrane proteins (Table S1).

###### Compartment-specific localization scores.

The sum of RPKM values obtained from TG particles, ER particles, and the cytosol was considered as total cytoplasmic mRNA expression. For each gene, the mean compartment-specific RPKM value was divided by the total cytoplasmic mRNA expression. As a result, each gene is assigned three localization scores that correspond to the fraction of its transcripts that localize to each of the three compartments: TGs, the ER, and the cytosol.

###### Compartment-specific enrichment of mRNAs that encode membrane/secretory proteins.

We considered an mRNA to be ER-enriched if the ratio of localization scores (ER/TG) was greater than 1.25 and classified it as TG-enriched if it was smaller than 0.8. The median localization score of membrane/secretory mRNAs in the cytosol was 0.09. If the cytosolic localization score of an mRNA was greater than 0.36, it was considered enriched in the cytosol. If the ER and TG-specific localization scores were similar and the cytosolic partition coefficient was smaller than 0.18, the mRNA was assigned to the ER, whereas it was considered not localized if the cytosolic localization score was smaller than 0.18 (Figure S1H).

###### Compartment-specific enrichment of mRNAs that encode non-membrane proteins.

To faithfully compare differences in mRNA distribution across the three compartments, it is necessary to know the relative size distribution of the three compartments. However, this parameter is currently unknown. Therefore, instead of comparing the localization scores across samples, we determined the most enriched mRNAs within each compartment. We considered an mRNA compartment-enriched, if its average localization score (from biological replicates) was at least 1.25-fold higher than the median localization score of its corresponding compartment samples. For TG particles, the median localization score was 0.32, for ER particles, it was 0.30, and for the cytosol, the median localization score was 0.34. If the enrichment was observed in two compartments, the mRNA was assigned to the compartment with the higher value. With this strategy, we identified 1246 TG+ mRNAs, 919 non-overlapping ER+ mRNAs, and 1481 CY+ mRNAs. The remaining 3369 mRNAs (48%) do not have a compartment-biased mRNA localization pattern and were called (unbiased).

Justification of the cut-off used to determine compartment-enriched mRNAs. A minimum cut-off of 1.25-fold higher than the median localization score corresponds to approximately one standard deviation. The compartment-enriched mRNAs differed substantially in their functional and architectural features (Figure 2). We generated subgroups among the compartment-enriched mRNAs that represent the top, middle, and bottom-enriched subgroups (Figure S4). Even when focusing on the bottom-enriched groups (which are close to the cut-off used), the differences in functional and architectural features across the compartment-enriched groups were still highly significant (Figure S4). The cut-off is further justified as we were able to validate 10/11 mRNAs considered to be compartment enriched with an independent method. Moreover, we demonstrate that TG-translated MYC has biological effects, despite *MYC* mRNA being found in the bottom enriched TG+ group.^19^

##### mRNA transcript distribution in HEK293T TIS11B KO cells

We focused on the analysis of mRNAs that encode non-membrane proteins (Table S5). The mean RPKM values of the biological replicates of digitonin-extracted samples and the ER particles were calculated for TIS11B KO cells and their corresponding control HEK293T cells. A gene was considered expressed if the average RPKM value in the ER and in the cytosol samples was greater than 3 RPKM (*N* = 6229). The compartment-specific localization scores were calculated and the difference in localization scores between TIS11B KO and control samples were calculated for ER and cytosol. The top 20% of genes with a localization change towards ER or the cytosol were intersected with genes considered as TG+ (*N* = 1246) and further analyzed with respect to their bound RBPs and architectural features.

##### mRNA and protein features of the localized mRNAs

RPKM values of mRNAs were obtained from RNA-seq data of unfractionated HEK293T cells and were determined for the compartment-biased mRNAs. Pro-seq and RNA-seq from HEK293 cells were obtained from GEO (GSE140365: PRO-seq; GSE142895: RNA-seq).^27^ Raw reads were processed by trimmomatic (version: 0.39) to trim low-quality ends (average quality per base < 15, 4 bp window) and adapters.^62^ Trimmed reads were mapped to the human genome (hg19) using hisat2 (version: 2.1.0).^63^ Reads mapped to each gene were counted by featureCounts (version: 1.6.4).^64^ To estimate mRNA stability rates, log_2_-normalized counts of Pro-seq data were divided by the log_2_-normalized RNA-seq data, as described previously.^28^ 3′UTR length of each mRNA was obtained from Ref-seq. The longest 3′UTR isoform of each gene is reported. mRNA length, CDS length, average CDS exon length, and total exon number of genes were determined using transcripts from the Matched Annotation from the NCBI and EMBL-EBI (MANE)^65^ human version 1.2. For each gene, the transcript with longest mRNA length was selected. Protein length was calculated by dividing CDS length by three.

##### Proteomics protein expression analysis

Protein expression was obtained from TMT-based quantitative mass spectrometry analysis of HEK293T cells. Precursor protein abundance was calculated for each protein and scaled to the TMT abundance for each channel. Relative abundance was then calculated by averaging the condition-specific biological replicates. In brief, mass spectra were processed using Protein Discoverer 2.5 (ThermoFisher) using the Minora algorithm (set to default parameters) for precursor quantification and using a TMTpro workflow for TMT-based quantification. Database searching included all canonical entries from the human Reference Proteome UniProt database (SwissProt – 2022–03), as well as an in-house curated list of contaminants. The identification of proteins was performed using the SEQUEST-HT engine against the database using the following parameters: a tolerance level of 10 ppm for MS^1^ and 0.6 Da for MS^2^ post-recalibration and the false discovery rate of the Percolator decoy database search was set to 1%. Trypsin was used as the digestion enzyme, two missed cleavages were allowed, and the minimal peptide length was set to 7 amino acids. Carbamido-methylation of cysteine residues (+57.021 Da) was set as static modifications, while oxidation of methionine residues (+15.995 Da) was set as a variable modification. The final protein-level FDR was set to 1%. Precursor abundance quantification was determined based on intensity, and the minimum replicate feature parameter was set at 50%. Proteins were quantified based on unique and razor peptides and proteins with less than two different peptides were excluded. For TMT-based quantification, similar search parameters were used, with the addition of TMTpro tags on lysine residues and peptide N termini (+304.207 Da) set as static modifications. For TMTpro-based reporter ion quantitation, the summed signal-to-noise (S:N) ratio for each TMT channel was extracted, and the closest matching centroid to the expected mass of the TMT reporter ion was found (integration tolerance of 0.003 Da). PSMs with poor quality, MS^3^ spectra with TMT reporter ion channels missing, or isolation specificity less than 0.7, or with less than 70% of SPS masses matching to the identified peptides, or with an average TMT reporter summed signal-to-noise ratio that was less than 10 or had no MS^3^ spectra were excluded from quantification. We exported the results of protein identification and quantification to Excel, including the TMT-based reporter ion quantitation. Additionally, we extracted the MS^1^ precursor abundance for each protein (Minora algorithm), which indicates its relative abundance in the tryptic sample. Each MS^1^-based abundance measured should be a representation of the sum of all the respective TMT-labeled peptides combined. Therefore, for a rudimentary metric of protein abundance across samples, we divided the total MS^1^-abundance for individual proteins by their respective TMT summed signal-to-noise ratio to each TMT channel.

##### CLIP data analysis

###### iCLIP analysis of TIS11B in HEK293T cells.

Raw fastq files were demultiplexed using the iCount python package (https://icount.readthedocs.io). 5′ and 3′ adapters were trimmed by Cutadapt.^66^ Trimmed reads were mapped to human genome using STAR and reads mapping to tRNA/rRNA were discarded.^67^ Crosslink sites were called from bam files using the ‘‘xlsites’’ function of iCount. CLIP-seq analysis was carried out on the iMaps platform (https://imaps.genialis.com/iclip), where peak calling was performed by analysing cDNA counts at crosslink sites using Paraclu.^68^ Motif analysis was carried out using HOMER software. Enrichment was calculated within the genomic coordinates of a total of 57,714 TIS11B CLIP peaks found in 3′UTRs. Total peaks: 190,920; peaks in 3′UTRs: 57,714.

###### POSTAR3 CLIP data.

CLIP data on 168 RBPs were downloaded from Postar3^34^ and peak counts that overlapped with annotated 3′UTRs from Ref-seq in all mRNAs that encode non-membrane proteins were recorded. For each RBP, the median number of 3′UTRs CLIP peaks was calculated and all 3′UTRs with peaks counts greater than the median were considered as targets. Based on the fraction of mRNAs that are considered compartment-specific (TG: 17.8%; ER 13.1%; CY: 21.1%; unbiased: 48.0%), we determined the expected number of target genes for each compartment. If the observed number of targets divided by the expected number of targets in a compartment was greater than 1.5, the RBP was added to our short-list (Table S4). As TIS11B and TIA1/L1 are known to bind to AU-rich sequences, we added the processed PAR-CLIP data of the LARP4B RBP as it was reported to bind to AU-rich elements.^33^

###### Logistic regression.

The R package ‘nnet’ (v7.3–17) was used to fit logistic regression models to predict the subcytoplasmic mRNA localization of non-membrane proteins. An initial model used CLIP peak counts from the RBPs on the short list (*N* = 24). A second model used the top seven RBPs from the first model fit and added mRNA length and average CDS exon length. Covariates with missing values were imputed as zeros. All covariates were first ‘sqrt’ transformed and then standardized. The ‘unbiased’ category was used as the base level. The R package ‘broom’ (v0.8.0) was used to compute t-test statistics for the model coefficients. The code is available on github (github.com/Mayrlab/tiger-seq).

###### Confirmation of the logistic regression.

To validate the contribution of each individual RBP, we used more stringent criteria to determine their targets. Among all mRNAs that encode non-membrane proteins with at least one CLIP peak in the 3′UTR, we considered the top third of mRNAs as targets of each RBP (TIS11B: 1781 targets; TIA1/L1: 1313 targets; LARP4B: 1621 targets; METAP2: 256 targets; HuR: 1124 targets; PUM2: 427 targets; HNRNPC: 232 targets). mRNAs only bound by LARP4B or METAP2 are LARP4B/ METAP2 targets and not bound by another RBP (from the seven RBPs investigated), *N* = 717. mRNAs predominantly bound by TIS11B are TIS11B targets exclusively bound by TIS11B or co-bound by TIA1/L1, with TIS11B/TIA1/L1 ≥ 2 (*N* = 834). mRNAs pre-dominantly bound by TIA1/L1 are TIA1/L1 targets exclusively bound by TIA1/L1 or co-bound by TIS11B but TIS11B/TIA1/L1 < 2 (*N* = 634).

##### Intersection of membrane/secretory mRNAs with previous datasets

###### APEX-seq.

The mRNAs that are coexpressed in our RNA-seq dataset (*N* = 9155 mRNAs) and the ER membrane-localized mRNAs from the APEX-seq dataset (*N* = 1045) were determined.^11^ The overlapping 845 mRNAs were intersected with the mRNAs that encode membrane/secretory proteins found to be ER+ in our analysis (*N* = 1476). We detected 673 mRNAs which correspond to 80% of all APEX-seq mRNAs that are considered to be ER membrane (ERM)-enriched. The universe used to test for enrichment were all mRNAs that encode non-membrane proteins (*N* = 2140).

###### Biochemical fractionation.

A similar analysis was performed for the fractionation dataset from Reid and Nicchitta.^9^ Among the 385 coexpressed mRNAs that are enriched on the ER according to Reid, we detected 308 in our ER+ fraction when focusing on membrane/secretory protein encoding mRNAs. This group represents 80% of all ER-enriched mRNAs detected by Reid.

###### MERFISH.

In the MERFISH dataset, which was generated in U2OS cells, 1037 mRNAs are considered ER-enriched. Among them, *N* = 571 are co-expressed in our dataset and considered mRNAs encoding membrane/secretory proteins. Among the 571 co-expressed mRNAs we consider 511 as ER+, which corresponds to 89%. Among the ER-de-enriched mRNAs (Log2FC nonER vs ER = −0.34), only 69 mRNAs encode membrane/secretory proteins. Among the 69 mRNAs, we consider 8 as ER+, which corresponds to 11.6%.^13^

###### Intersection of mRNAs that encode non-membrane proteins with a previous dataset

The relative distribution of mRNA transcripts across subcellular compartments, including the membrane fraction, phase-separated granules, and the cytosol was determined using density gradient centrifugation in U2OS cells.^25^ The number of co-expressed mRNAs that encode non-membrane proteins was *N* = 6557, which corresponds to 93% of our dataset. This dataset determines the proportion of transcripts that localize to the different fractions. For co-expressed TG+ mRNAs (*N* = 1153), ER+ mRNAs (*N* = 839) and CY+ mRNAs (*N* = 1400), we plotted the proportion of mRNAs that localize to phase-separated granules, to the membrane fraction, and to the cytosol in the LoRNA dataset in U2OS cells.

###### Gene ontology analysis

Gene ontology (GO) analysis was performed using DAVID.^30^

### QUANTIFICATION AND STATISTICAL ANALYSIS

Statistical parameters are reported in the figures and figure legends, including the definitions and exact values of *N* and experimental measures (mean ± SD or boxplots depicting median, 25^th^ and 75^th^ percentile (boxes) and 5% and 95% confidence intervals (error bars). Pair-wise transcriptomic feature comparisons were performed using a two-sided Mann-Whitney test. For more than two samples, a Kruskal-Wallis test was performed. For transcriptomic analyses, statistical significance is indicated by asterisks *, 0.05 > P > 1 × 10–9; **, 1 × 10–10 > P > 1 × 10–20; ***, 1 × 10–21 > P > 1 × 10–80; ****, 1 × 10–81 > P > 0. Exact P values are listed in Table S3. Enrichment was determined using a X^2^ test. The *P* value was calculated using a two-sided Fisher’s exact test. When indicated, a two-sided t-test with assumption of equal variance was applied. Statistical significance for experimental data is indicated by asterisks *, *P* < 0.05, **, *P* < 0.01, ***, *P* < 0.001, ****, *P* < 0.0001.

## Supplementary Material

MMC6

MMC4

MMC3

MMC5

MMC2

MMC1

## Figures and Tables

**Table:** KEY RESOURCES TABLE

REAGENT or RESOURCE	SOURCE	IDENTIFIER

**Antibodies**		
Rabbit anti-GFP	Abcam	Cat# ab290, RRID:AB_303395
Rabbit anti-TIS11B	Cell Signaling	Cat# 30894
Mouse anti-GAPDH	Sigma-Aldrich	Cat# G8795, RRID:AB_1078991
Rabbit anti-Calnexin	Abcam	Cat# ab22595, RRID:AB_2069006
Rabbit anti-H3	Cell Signaling	Cat# 9715, RRID:AB_331563
Chicken anti-GFP	Abcam	Cat# Ab13970, RRID:AB_300798
Donkey anti-mouse IRDye 700	Rockland	Cat# 610-730-002, RRID:AB_1660934
Donkey anti-rabbit IRDye 800	LI-COR Biosciences	Cat# 926-32213, RRID:AB_621848
Donkey anti-mouse IRDye 800	LI-COR Biosciences	Cat# 926-32212, RRID:AB_621847

**Chemicals, peptides, and recombinant proteins**		
Stellaris^®^ FISH Probes, eGFP with Quasar^®^ 670 Dye	Biosearchtech	Cat# VSMF-1015-5
Lipofectamine 3000	ThermoFisher	Cat# L3000001
Chloroquine diphosphate salt	Millipore-Sigma	Cat# 6628
Fibronectin	Millipore-Sigma	Cat# F0635
Odyssey blocking buffer (PBS)	LI-COR Biosciences	Cat# 927-40000
SeeBlue Plus2 Pre-Stained Standard	Thermo Fisher Scientific	Cat# LC5925
NuPAGE MES SDS running buffer 20x	Invitrogen	Cat# NP0002
NuPAGE Novex 4-12% Bis-Tris Protein Gels, 1.0 mm, 10 well	Invitrogen	Cat# NP0321
NuPAGE Transfer Buffer	Invitrogen	Cat# NP00061
Sample Buffer, Laemmli 2× Concentrate	Sigma-Aldrich	Cat# S3401
Tween-20	Fisher Scientific	Cat# BP337-500
Triton X-100	Fisher Scientific	Cat# BP151-100
Nonidet P-40	Sigma-Aldrich	Cat# 74385
IGEPAL CA-630	Millipore-Sigma	Cat# I8896
Doxycycline Hydrochloride	Millipore-Sigma	Cat# D3447
Ampicillin Sodium Salt	Fisher Scientific	Cat# BP176025
Puromycin Dihydrochloride	Fisher Scientific	Cat# A1113803
Bovine Serum Albumin (BSA)	Fisher Scientific	Cat# BP1605100
Tris Base	Fisher Scientific	Cat# BP152-1
Sodium Chloride	Fisher Scientific	Cat# S271-3
Dextran Sulfate Sodium Salt	Spectrum Chemical	Cat# DE131
Dextran Sulfate 50% solution	Millipore-Sigma	Cat# S4030
Ethylene Carbonate, 98%	Millipore-Sigma	Cat# E26258-500G
Ribonucleoside Vanadyl Complex	NEB	Cat# S1402
Salmon testes single stranded DNA	Sigma-Aldrich	Cat# D7656
Yeast tRNA	Life Technologies	Cat# 15401029
Formamide	Sigma-Aldrich	Cat# F7503
Murine RNase Inhibitor	NEB	Cat# M0314S
AMPure XP	Fisher Scientific	Cat# NC9959336
Protein A/G Magnetic Beads	ThermoFisher	Cat# 88802
Superscript IV Reverse Transcriptase	ThermoFisher	Cat# 18090010
DAPI (4’,6-Diamidino-2-Phenylindole, Dihydrochloride)	Life Technologies	Cat# D1306
Quant-it Ribogreen RNA Reagent	ThermoFisher	Cat# R11491
TRI Reagent^™^ Solution	Invitrogen	Cat# AM9738
SuperScript IV VILO Master Mix with ezDNase	Invitrogen	Cat# 11766050
Q5 High-Fidelity DNA Polymerase	NEB	Cat# M0491L
T4 DNA Ligase	NEB	Cat# M0202L
DNA Polymerase I, Large (Klenow) Fragment	NEB	Cat# M0210L
UltraPure agarose	Invitrogen	Cat# 16500500
Ethidium Bromide	Fisher Scientific	Cat# PI17898
16% Paraformaldehde Aqueous Solution	Fisher Scientific	Cat# 50-980-487
ProLong Gold Antifade Mountant	ThermoFisher	Cat# P36934
ProLong Diamond Antifade Mountant	ThermoFisher	Cat# P36961
Methanol	Fisher Scientific	Cat# A412-4
Ethanol	Fisher Scientific	Cat# BP28184
Isopropanol	Fisher Scientific	Cat# BP26184
Chloroform	Fisher Scientific	Cat# C607-4
Urea	Sigma Aldrich	Cat# U0631
EPPS	Sigma Aldrich	Cat# E9502-1KG
cOmplete EDTA-free Protease Inhibitor Cocktail	Roche	Cat# 04-693-159-001
Phosphatase Inhibitor Cocktail 2	Sigma Aldrich	Cat# P5726
Phosphatase Inhibitor Cocktail 3	Sigma Aldrich	Cat# P0044
Benzonase	Sigma Aldrich	Cat# E8263-5KU
TCEP Solution	ThermoFisher	Cat# PI77720
Lysyl endopeptidase	FUJIFILM Wako	Cat# 129-02541
Sequencing Grade Modified Trypsin	Promega	Cat# V5111
Acetonitrile anhydrous	Sigma Aldrich	Cat# 271004
Hydroxylamine	Sigma Aldrich	Cat# 467804
Trifluoroacetic acid	Sigma Aldrich	Cat# T6508-10AMP
Ammonium biocarbonate BioUltra	Sigma Aldrich	Cat# 09830
Water, Optima LC/MS Grade	FisherScientific	Cat# W6-1
Formic acid	FisherScientific	Cat# A117-10X1AMP

**Critical commercial assays**		

QIAGEN Plasmid Plus Midi Kit	Qiagen	Cat# 12945
TMTpro 16plex Label Reagent Set	ThermoFisher	Cat# A44520

**Experimental models: Cell lines**		

HeLa	Jonathan S. Weissman	N/A
HEK293T	ATCC	ATCC Cat# CRL-3216, RRID:CVCL_0063
Oligonucleotides	This paper	Table S6

**Recombinant DNA**		

pcDNA-GFP-TIS11B	Ma and Mayr^16^	N/A
pcDNA-BFP-TIS11B	Ma and Mayr^16^	N/A
pcDNA-mCherry-TIS11B	Ma and Mayr^16^	N/A
pcDNA-GFP-SEC61B	Ma and Mayr^16^	N/A
pcDNA-BFP-SEC61B	Ma and Mayr^16^	N/A
pcDNA-mCherry-SEC61B	Ma and Mayr^16^	N/A
pcDNA-GFP	Ma and Mayr^16^	N/A
pcDNA-mCherry	Ma and Mayr^16^	N/A
pcDNA-BIRC3-MS2-SU	Lee and Mayr^39^	N/A
pFRT_TO_FlagHA_TIAL1	Meyer et al.^36^	Addgene106090
UBC NLS-HA-2XMCP-tagRFPt	Halstead et al.^52^	Addgene 64541
pHR-tdPP7-3xmCherry	Yan et al.^32^	Addgene 74926
pHR-scFv-GCN4-sfGFP-GB1-dWPRE	Yan et al.^32^	Addgene 60907
pcDNA4TO-24xGCN4_v4-kif18b-24xPP7	Yan et al.^32^	Addgene 74928
pcDNA-GFP-THAP1-MS2	This paper	N/A
pcDNA-MCP-mCherry	This paper	N/A
pcDNA-MCP-mCherry-TIS11B	This paper	N/A
pcDNA-MCP-mCherry-HuR	Lee and Mayr^39^	N/A
pcDNA-MCP-mCherry-TIAL1	This paper	N/A
pcDNA-MCP-mCherry-SEC61B	This paper	N/A
pcDNA-TRAPa-MCP-mCherry	This paper	N/A
pcDNA-MCP-mCherry-CAAX	This paper	N/A
pcDNA-MCP-mCherry-TIAL1-CAAX	This paper	N/A
pcDNA-TIS11B-MCP-mCherry-SEC61B	This paper	N/A

**Software and algorithms**		

FIJI	NIH	https://fiji.sc/
MATLAB	MATLAB	https://www.mathworks.com/products/matlab.html
HOMER	UCSD	http://homer.ucsd.edu/homer/
GraphPad Prism 8	GraphPad Software	https://www.graphpad.com/scientific-software/prism
FlowJo_V10	FlowJo	https://www.fiowjo.com
SPSS Software Version 14	IBM SPSS Statistics	https://www.ibm.com/products/spss-statistics
R Studio	R Project	https://www.r-project.org/
Odyssey	LI-COR Biosciences	https://www.licor.com/bio/products/imaging_systems/odyssey/

**Deposited data**		

Raw data	This paper	https://doi.org/10.17632/nmt7ppsp8r1
Image analysis scripts	This paper	https://doi.org/10.17632/nmt7ppsp8r1
RNA-seq datasets	This paper	GEO, Accession number: GSE215770
Proteomics dataset	This paper	MassIVE repository (sitory (dataset identifier MSV000092176)
Original code for data analysis	This paper	https://doi.org/10.5281/zenodo.10056230

## Highlights

- The majority of genes generate transcripts with biased subcytoplasmic localization
- Gene architecture and RNA-binding proteins influence cytoplasmic mRNA localization
- mRNAs that encode transcription factors are strongly enriched in TIS granules
- A change in mRNA localization changes protein abundance
