# Supplementary material for: Subcytoplasmic location of translation controls protein output
Source: Mol Cell. Author manuscript; Available in PMC 2024 Jun 3. (PMC11146010; doi:10.1016/j.molcel.2023.11.025)
Supplement: MMC1 [file NIHMS1953329-supplement-MMC1.pdf]

Molecular Cell, Volume *83*

Supplemental information

Subcytoplasmic location of translation controls protein output

Ellen L. Horste, Mervin M. Fansler, Ting Cai, Xiuzhen Chen, Sibylle Mitschka, Gang Zhen, Flora C.Y. Lee, Jernej Ule, and Christine Mayr

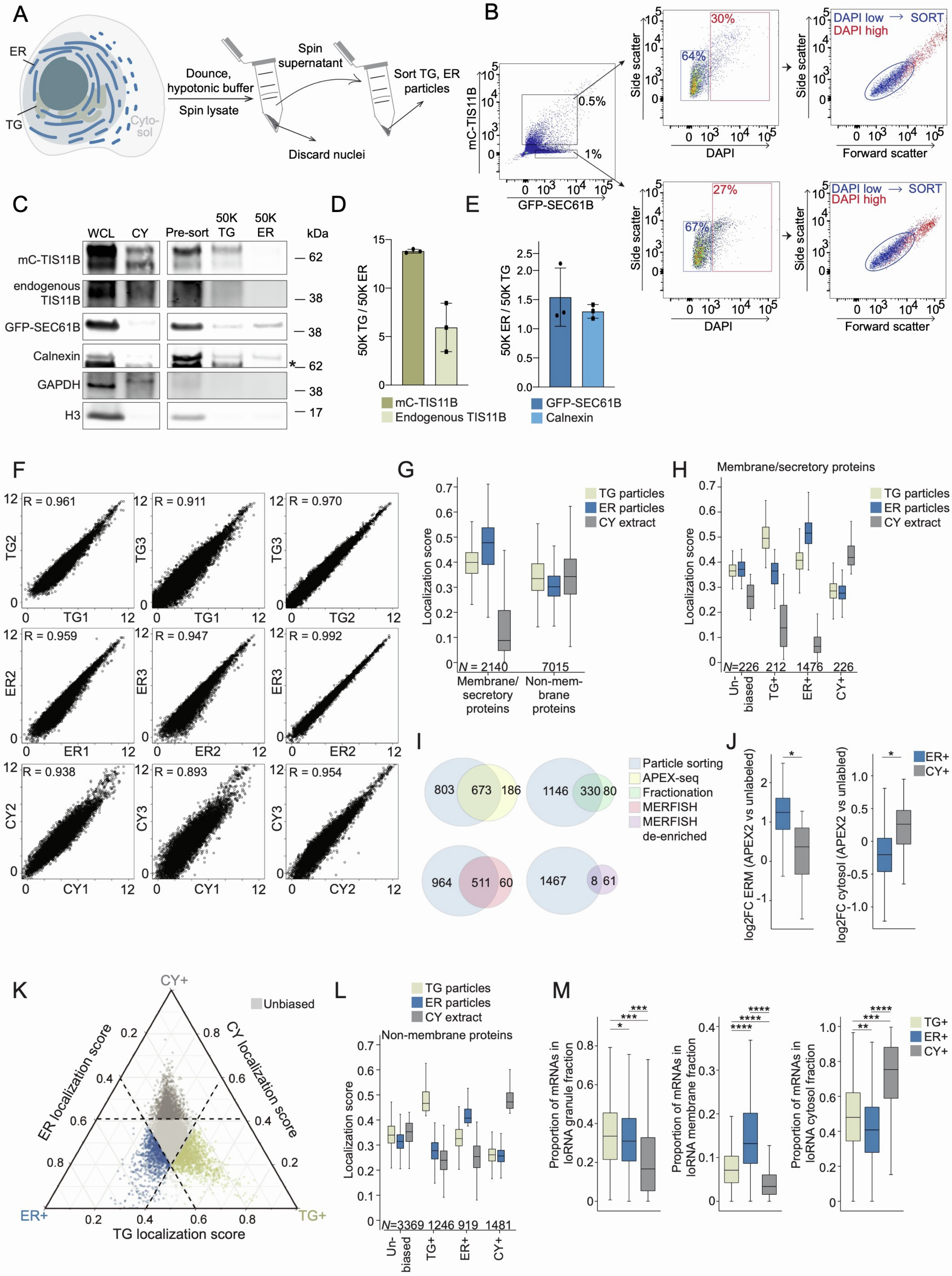

Figure S1. Strategy to determine subcytoplasmic mRNA localization, related to Figure 1.

Figure S1. Strategy to determine subcytoplasmic mRNA localization, related to Figure 1.

(A) Cell fractionation strategy to obtain the cytoplasmic membrane fraction. Transfected HEK293T cells were lysed in hypotonic buffer, followed by douncing and differential centrifugation at 600 g to pellet nuclei. The supernatant was subsequently spun at 7000 g. The pellet contains the cytoplasmic membrane fraction that was used for subsequent fluorescent particle sorting.

(B) FACS plot showing the gating strategy to obtain mCherry-TIS11B+/GFP-SEC61B+ TG particles and mCherry-TIS11B−/GFP-SEC61B+ ER particles. The particles were costained with DAPI to segregate TG and ER particles from nuclear contamination (DAPI high). The DAPI low particles were sorted. For subsequent RNA-seq experiments, we sorted TG particles from the TIS11B+SEC61B+ population and we sorted ER particles from the TIS11B−SEC61B+ population.

(C) Immunoblot showing markers used to evaluate the quality of the three compartment fractions. WCL is the unfractionated whole cell lysate. CY is the digitonin-extracted cytosol. Pre-sort is the membrane-enriched cytoplasmic lysate from which TG and ER particles are sorted, which is the final step in (A). 50K TG indicates 50,000 sorted TG particles and 50K ER indicates 50,000 sorted ER particles. H3 antibody was used as a marker for nuclear components, GAPDH was used as marker for cytosolic proteins, and Calnexin and GFP-SEC61B were used as ER markers. *, signal from mCherry-TIS11B. We modestly overexpress mCherry-TIS11B compared to its endogenous levels, which results in approximately 30% of cells to form TGs. This amount was chosen, because 25-30% of HEK293T cells form TG from endogenous TIS11B.

(D) Quantification of endogenous TIS11B and mCherry-TIS11B from (C) together with additional biological replicates. Shown is the ratio of protein abundance in 50K TG over 50K ER as mean ± std of three independent experiments.

(E) As in (D), but quantification of endogenous Calnexin and GFP-SEC61B. Shown is the ratio of protein abundance in 50K ER over 50K CY as mean ± std of three independent experiments.

(F) Correlation of log2-transformed RPKM values obtained by RNA-seq for biological replicates of subcytoplasmic compartments. The Pearson correlation coefficients are shown.

(G) Baseline distribution of localization scores across the three investigated cytoplasmic compartments is shown separately for mRNAs that encode membrane/secretory proteins and mRNAs that encode non-membrane proteins.

(H) Distribution of localization scores in each fractionation sample for compartment-enriched mRNAs that encode membrane/secretory proteins.

(I) Overlap of ER+ mRNAs that encode membrane/secretory proteins (N = 1476) defined by us (particle sorting) with previous datasets that used alternative isolation methods. In the APEX-seq dataset 78% of ER-localized mRNAs overlap with our ER+ mRNAs. In the fractionation dataset, 80.5% of ER-localized mRNAs overlap with our ER+ mRNAs. Among the MERFISH ER-localized mRNAs 89% overlap with our ER+

mRNAs and only 11.6% of our ER+ mRNAs overlap with the ER-de-enriched mRNAs obtained by MERFISH.

(J) ER+ mRNAs that encode membrane/secretory proteins (N = 1476) defined by us are significantly enriched on the ER membrane (ERM) according to APEX-seq, whereas CY+ mRNAs that encode membrane/secretory proteins show a significantly lower ERM enrichment. Similarly, CY+ mRNAs that encode membrane/secretory proteins have significantly higher APEX2 enrichment scores in the cytosol than mRNAs considered ER+ by us. Mann Whitney test: $P < 2x10^{-7}$.

(K) Ternary plot showing compartment-enriched mRNAs. Each dot represents an mRNA that is color-coded as in Fig. 1D-F. mRNAs in the center (light grey) are considered to have an unbiased transcript distribution.

(L) Distribution of localization scores in each fractionation sample for compartment-enriched mRNAs that encode non-membrane proteins.

(M) Validation of mRNAs that encode non-membrane proteins and are defined as compartment-enriched by us in comparison with the LoRNA dataset. Our TG+ mRNAs are highest enriched in the mRNAs that LoRNA identifies in the phase-separated granule fraction. Our ER+ mRNAs are highest enriched in the mRNAs that LoRNA identifies in the membrane fraction. Our CY+ mRNAs are highest enriched in the mRNAs that LoRNA identifies in the cytosol. Mann Whitney test was performed. Exact P values are listed in Table S3.

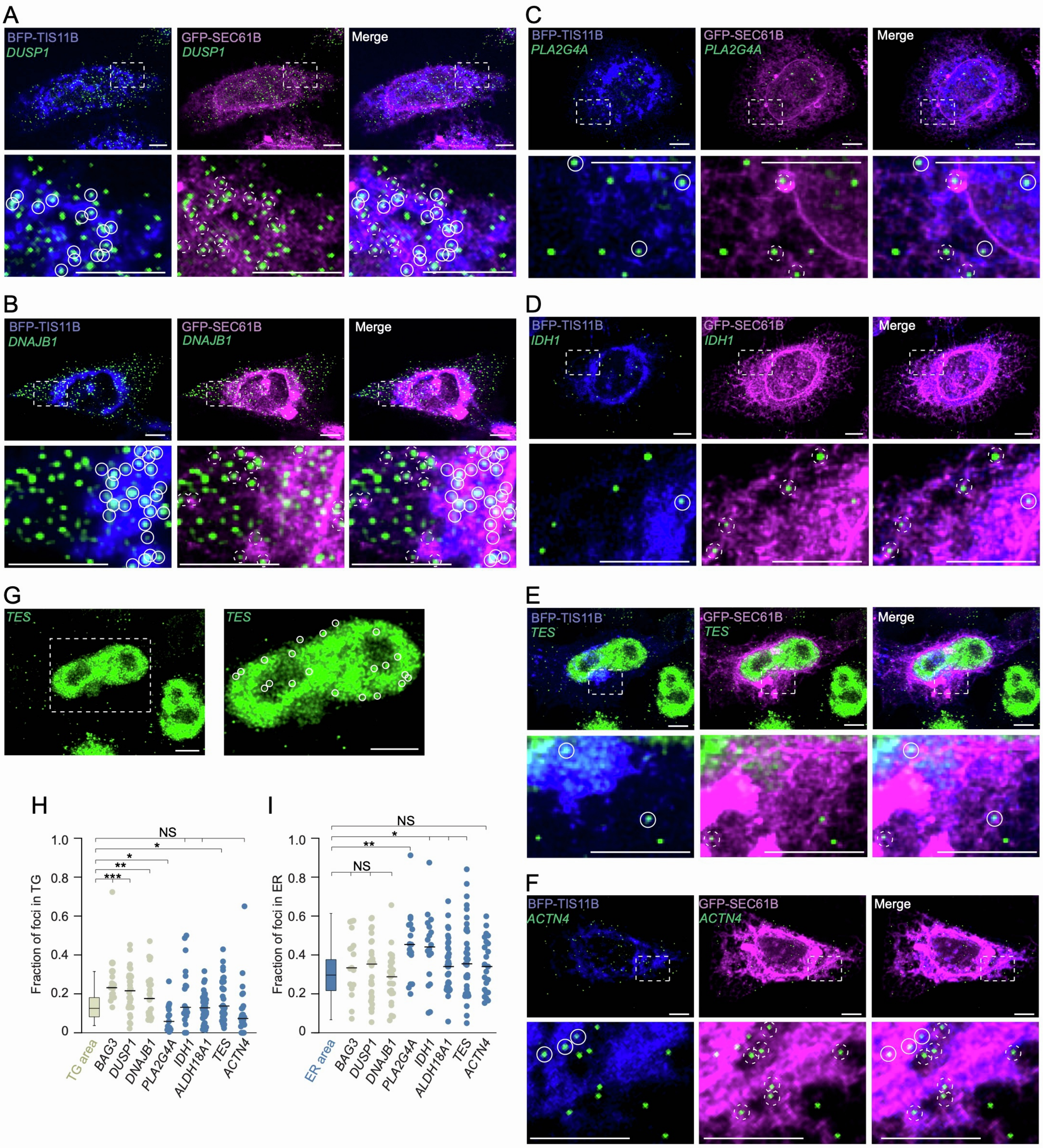

Figure S2. Validation of endogenous TG+ and ER+ mRNAs by smRNA-FISH, related to Figure 1.

(A) smRNA-FISH of endogenous TG+ mRNA DUSP1 (green) in HeLa cells. TGs (BFP-TIS11B, blue) and the ER (GFP-SEC-61B, magenta) were simultaneously visualized. The maximum projection of the fluorescent signals is shown. Bottom panel shows a 5x zoom-in of the area indicated by the white dashed box. White circles indicate colocalization of mRNA puncta with TGs, whereas dashed circles indicate colocalization with the ER. Representative images are shown. Scale bar, 5 μm.

Figure S2. Validation of endogenous TG+ and ER+ mRNAs by smRNA-FISH, related to Figure 1, continued.

(B) As in (A), but smRNA-FISH of endogenous TG+ mRNA *DNAJB1* is shown.

(C) As in (A), but smRNA-FISH of endogenous ER+ mRNA *PLA2G4A* is shown.

(D) As in (A), but smRNA-FISH of endogenous ER+ mRNA *IDH1* is shown.

(E) As in (A), but smRNA-FISH of endogenous ER+ mRNA *TES* is shown.

(F) As in (A), but smRNA-FISH of endogenous ER+ mRNA *ACTN4* is shown.

(G) As in (E) but shown is a 2x magnification of the area indicated by the white dashed box. The image illustrates the high background signal in the nucleus observed when probing for endogenous *TES*. For quantification of this mRNA, the noise tolerance value used in the puncta-calling function was increased to limit nuclear background noise. The white circles indicate nuclear foci that are included in the quantification at this tolerance level. Scale bar, 5 µm.

(H) Colocalization of TGs and smRNA-FISH foci of three TG+ (beige) and five ER+ (blue) endogenous mRNAs. The beige box plot indicates the expected fraction of mRNA transcripts based on the TG compartment size distribution, obtained from 186 cells. Number of cells analyzed for RNA-FISH: *BAG3* (*N* = 17), *DUSP1* (*N* = 30), *DNAJB1* (*N* = 25), *PLA2G4A* (*N* = 17), *IDH1* (*N* = 18), *ALDH18A1* (*N* = 22), *TES* (*N* = 34), *ACTN4* (*N* = 23). Mann Whitney test shows that the mRNA transcript distribution of 3/3 TG+ mRNAs (beige) is significantly higher than what would be expected for unbiased mRNAs. *P* value categories as in Figure 2A, exact *P* values listed in Table S3.

(I) As in (H), but colocalization of the ER and smRNA-FISH foci of three TG+ (beige) and five ER+ (blue) endogenous mRNAs. The blue box plot indicates the expected fraction of mRNA transcripts based on the ER compartment size distribution, obtained from of 186 cells. Mann Whitney test shows that the mRNA transcript distribution of 4/5 ER+ mRNAs (blue) is significantly higher than what would be expected for unbiased mRNAs.

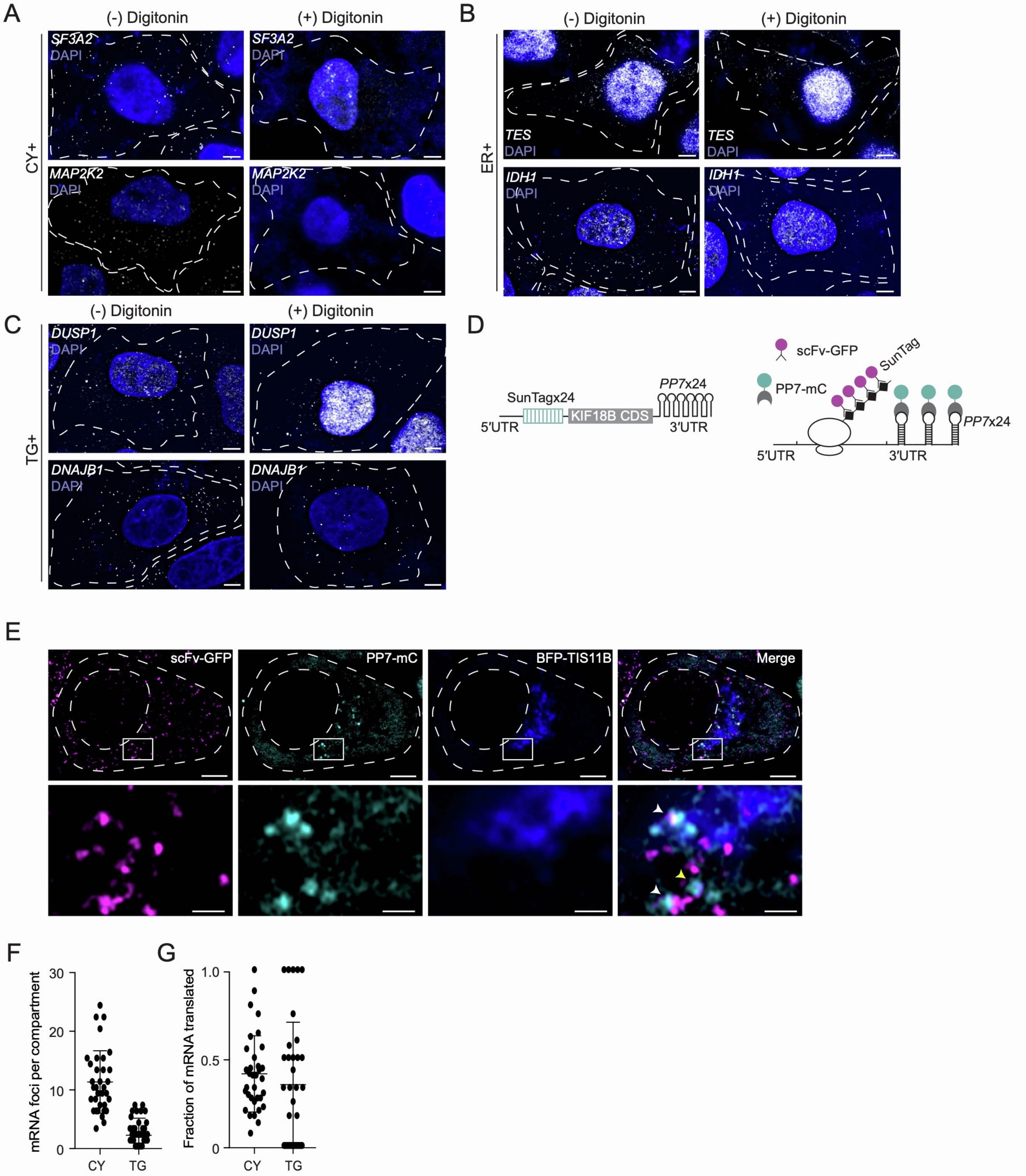

Figure S3. Validation of CY+ mRNAs by smRNA-FISH after digitonin extraction, related to Figure 1.

(A) Shown are smRNA-FISH foci of endogenous SF3A2 mRNA and MAP2K2 mRNA in HeLa cells before (-) and after (+) digitonin extraction. SF3A2 and MAP2K2 are considered CY+ mRNAs. Cell boundaries are indicated by the dotted lines. Representative images are shown. Scale bar, 5 μm.

(B) As in (A), but smRNA-FISH foci of endogenous TES mRNA and IDH1 mRNA are shown, which are considered ER+ mRNAs.

Figure S3. Validation of CY+ mRNAs by smRNA-FISH after digitonin extraction, related to Figure 1.

(C) As in (A), but smRNA-FISH foci of endogenous *DUSP1* mRNA and *DNAJB1* mRNA are shown, which are considered TG+ mRNAs.

(D) Schematic of the reporter mRNA used with the SunTag system to measure nascent protein synthesis. CDS, coding sequence. The KIF18B construct was used previously.[32]

(E) Confocal imaging of HeLa cells stably expressing SunTag reporter proteins svFc-GFP and mCherry-tagged PP7 protein (mC-PP7) co-transfected with two constructs (i) BFP-TIS11B to visualize TGs and (ii) SunTag-labeled mRNA encoding KIF18B with PP7-binding sites in the 3′UTR. The *KIF18B* mRNA is visualized by mC-PP7 binding (teal) whereas the KIF18B protein is visualized by svFc-GFP binding (magenta). Foci with co-localization of mRNA and protein represent nascent protein synthesis and are indicative of active translation. A representative example is shown. The white box indicated area is shown at 6x magnification in the lower panel. White arrows indicate actively translating mRNA, yellow arrow indicates non-translating mRNA. Scale bar, 5 μm (top panel), 1 μm (bottom panel).

(F) Quantification of the experiment from (E). Shown are the number of mRNA foci in TGs or the cytosol (CY) using the Suntag reporter from (D). Each dot represents a cell. *N* = 24 cells were analyzed.

(G) As in (F), but shown are the mRNAs that are actively translated in each compartment, which were identified by counting the teal and magenta-double positive foci.

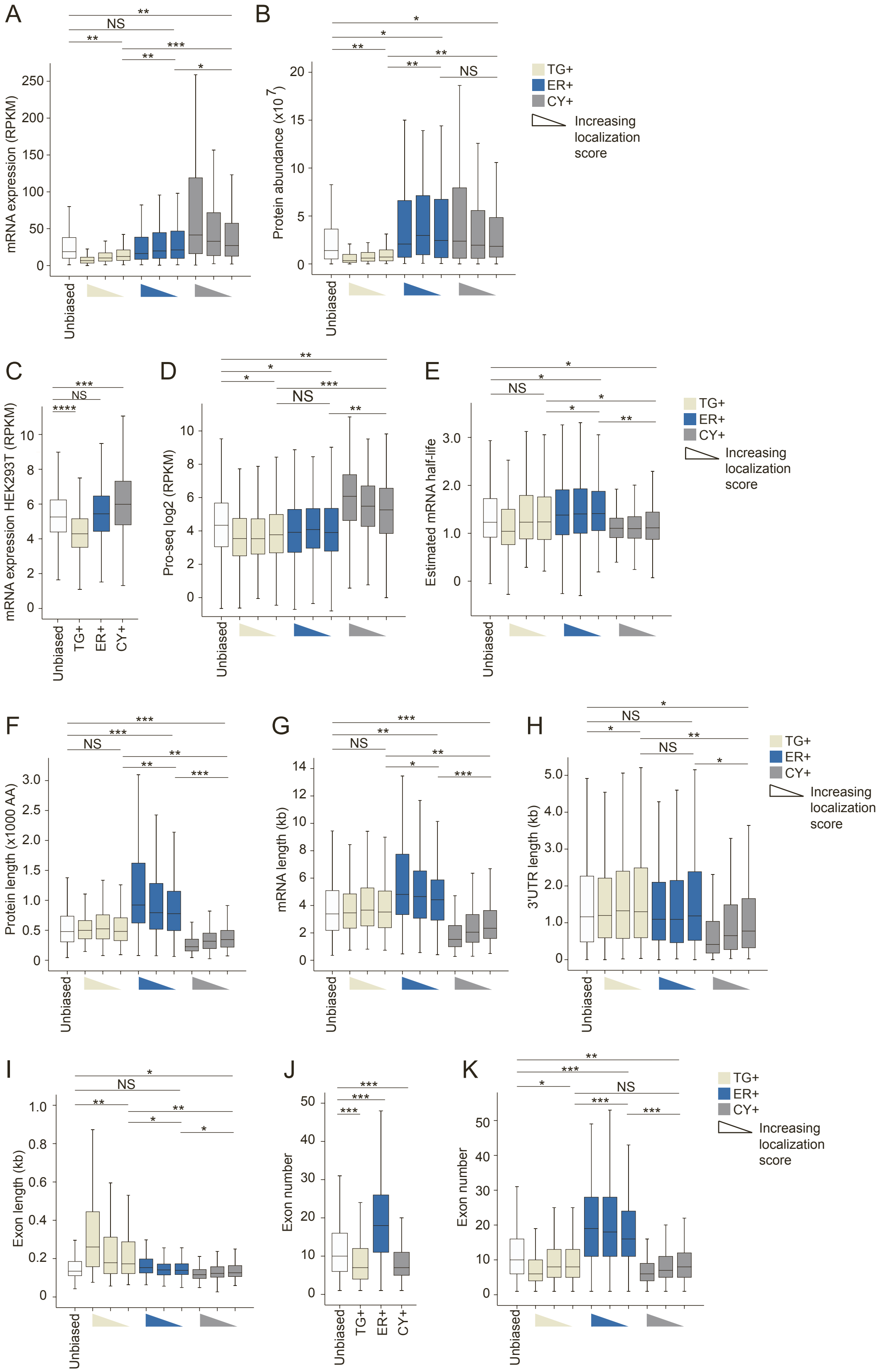

Figure S4. Characteristics of compartment-enriched mRNAs are shown for subgroups, related to Figure 2.

Figure S4. Characteristics of compartment-enriched mRNAs are shown for subgroups, related to Figure 2.

(A) To justify the cut-off used to determine compartment-enriched mRNAs (Fig. 1D-F), we show the data from Figure 2 in more detail. Steady-state mRNA abundance levels obtained from whole cell lysates is shown for unbiased mRNAs (*N* = 3369), three subgroups of TG+ mRNAs (top, middle, bottom, *N* = 415 each); three subgroups of ER+ mRNAs (top, middle, bottom, *N* = 306 each); three subgroups of CY+ mRNAs (top, middle, bottom, *N* = 493 each). Even when focusing on the bottom-enriched groups (which are close to the cut-off used), the differences across the compartment-enriched groups are still highly significant. Mann Whitney tests were performed. *P* value categories as in Fig. 2A. Exact *P* values are shown in Table S3. RPKM, reads per kilobase of transcript per million reads mapped.

(B) As in (A), but steady-state protein levels obtained from whole cell lysates are shown.

(C) As in Fig. 2C, but steady-state mRNA abundance levels of compartment-enriched mRNAs obtained by RNA-seq from whole cell lysates of HEK293 cells are shown. This sample was used together with the Pro-seq sample to estimate mRNA half-lives.

(D) As in (A), but Pro-seq levels are shown.

(E) As in (A), but estimated mRNA half-lives are shown.

(F) As in (A), but protein size distributions are shown.

(G) As in (A), but mRNA length distributions are shown.

(H) As in (A), but 3′UTR length distributions are shown.

(I) As in (A), but average CDS exon length distributions are shown.

(J) As in Fig. 2A, but the number of exons per mRNA is shown.

(K) As in (A), but the number of exons per mRNA is shown.

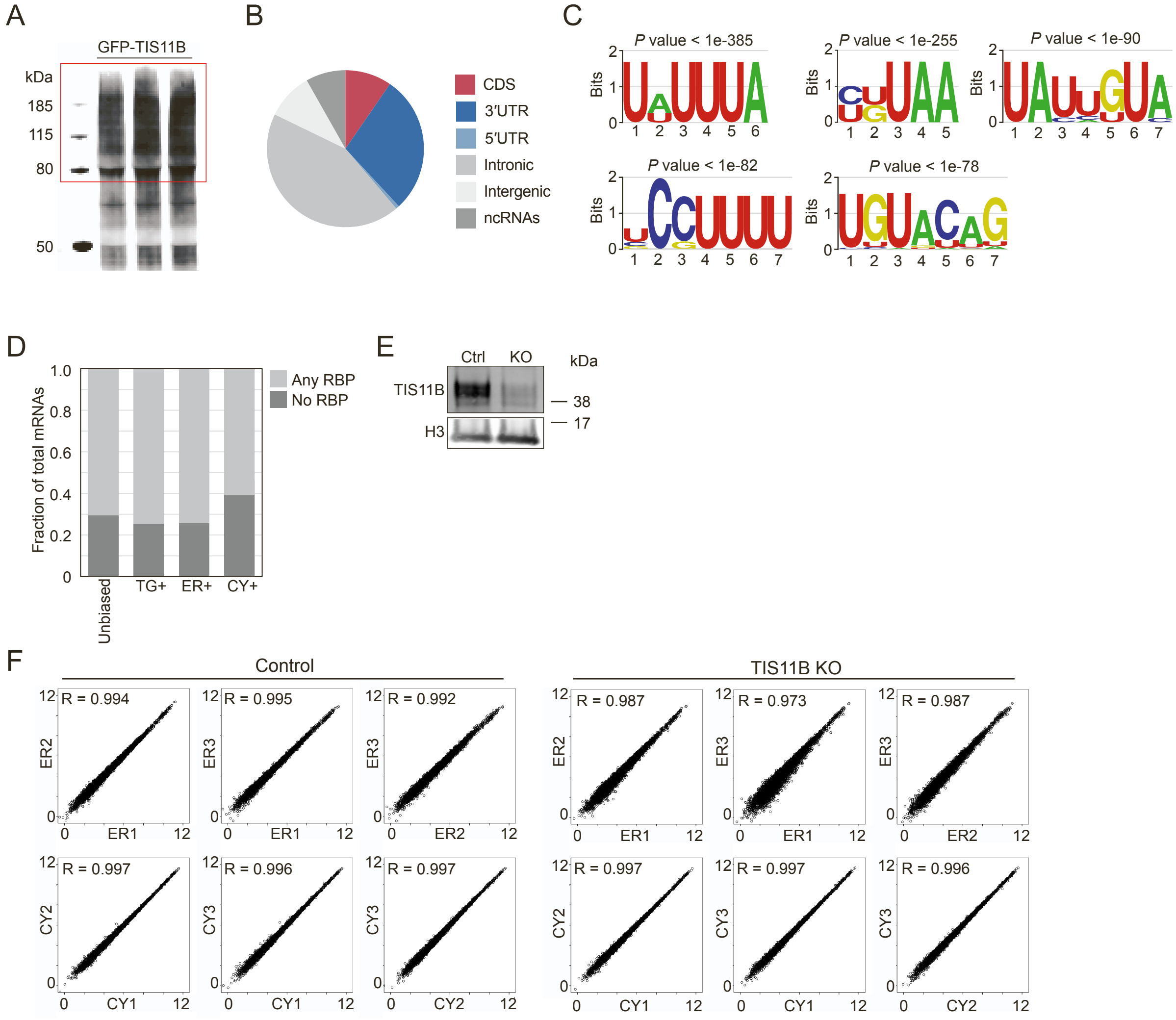

Figure S5. Analyses of TIS11B CLIP data and TIS11B KO samples, related to Figures 3 and 4.

(A) Gel showing samples used for iCLIP of GFP-tagged TIS11B. The region outlined in red was used for iCLIP sample preparation.

(B) TIS11B iCLIP tag distribution obtained from HEK293T cells.

(C) The top five motifs that were enriched within TIS11B peaks in 3′UTRs compared to all nucleotides in 3′UTRs. Shown are P values obtained by HOMER.

(D) The fraction of mRNAs bound by at least one RBP (from Fig. 3A) for the different groups of compartment-enriched mRNAs is shown.

(E) Immunoblot of TIS11B in control cells and TIS11B KO HEK293T cells. H3 was used as loading control.

(F) Correlation of log2-transformed RPKM values obtained by RNA-seq for biological replicates of sorted ER particles or digitonin-extracted cytosol samples for control and TIS11B KO cells. The Pearson correlation coefficients are shown.

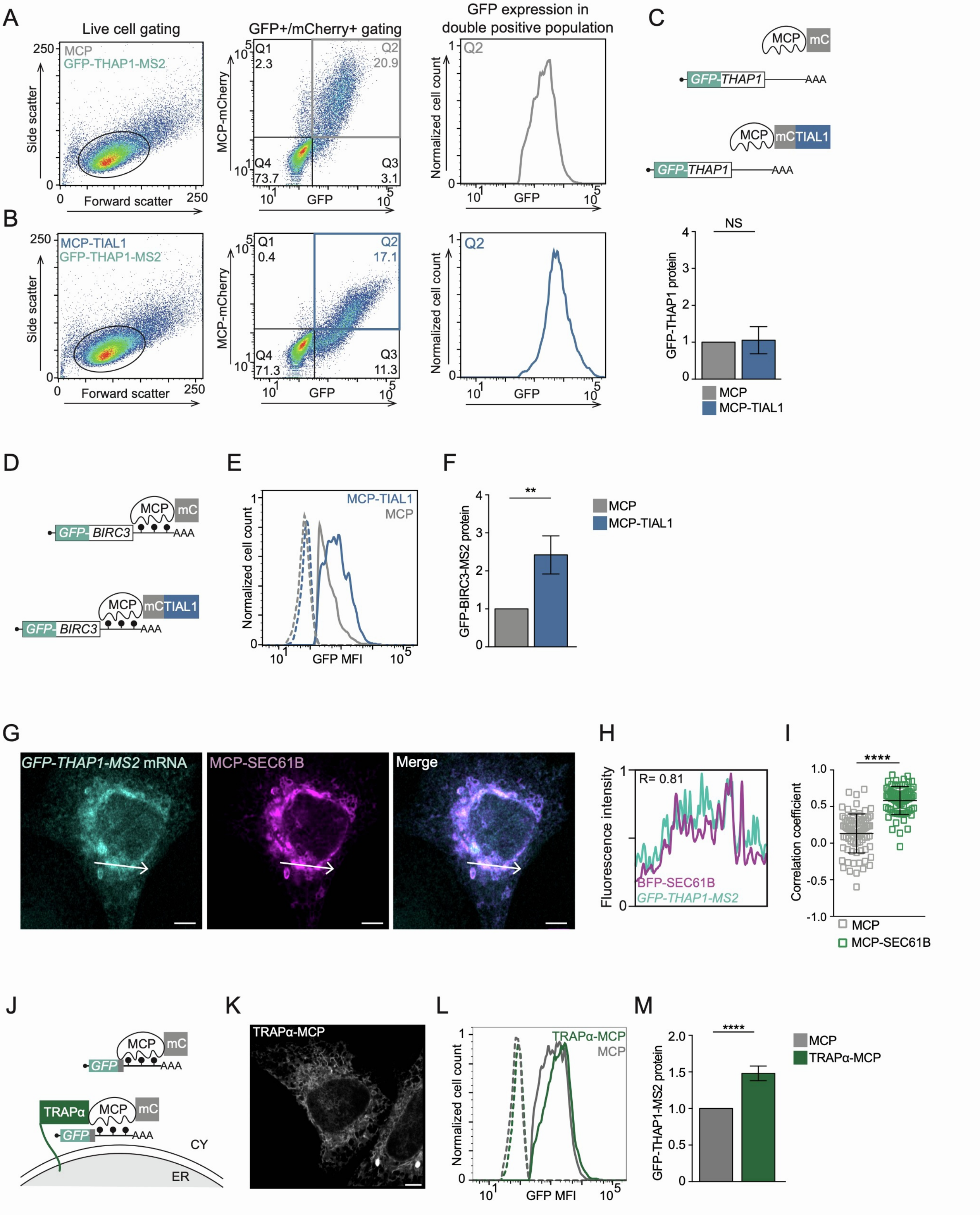

Figure S6. mRNA localization-dependent protein expression of the GFP reporter, related to Figure 5.

Figure S6. mRNA localization-dependent protein expression of the GFP reporter, related to Figure 5.

(A) Gating strategy to assess GFP protein expression of the reporter mRNA by FACS. Left panel shows the ungated population of HeLa cells coexpressing MCP-mCherry and the *GFP-THAP1-MS2* reporter, separated by size (forward scatter) and granularity (side scatter). The black circle indicates the live cells that were used for subsequent analysis. Middle panel, the GFP- and mCherry-double positive population was gated to obtain the GFP mean fluorescence values (MFI, right panel) which corresponds to the reported GFP protein expression values.

(B) As in (A), but HeLa cells coexpressing MCP-mCherry-TIAL1 and the *GFP-THAP1-MS2* reporter.

(C) As in Fig. 4F and 5C, but the MS2 sites in the GFP reporter were omitted. Top: coexpression of MCP-mCherry-TIAL1 does not result in the binding of MCP to the reporter mRNA without MS2 sites. This experiment serves as control for the effect of TIA1L1 overexpression on reporter mRNA expression. Bottom: Quantification of the experiment with the GFP mRNA reporter lacking the MS2 binding sites. Shown is the mean ± std of three independent experiments. T-test for independent samples, NS, not significant.

(D) Schematic of a second mRNA reporter used to validate the effect of a single 3′UTR-bound RBP on protein expression. The *GFP-BIRC3* reporter mRNA contains the BIRC3 coding region and MS2 hairpins as 3′UTR, which allow binding of the co-transfected MS2 coat protein (mCherry-tagged MCP). Fusion of TIAL1 to MCP tethers TIAL1 to the 3′UTR of the reporter mRNA. mC, mCherry.

(E) GFP protein expression of the reporter mRNA from (D) in HeLa cells, coexpressing the indicated MCP-fusion constructs, measured by FACS. Representative histograms are shown. GFP-negative cell populations are shown as dotted lines.

(F) Quantification of the experiment shown in (E). Shown is the mean ± std of five independent experiments. T-test for independent samples, **, $P = 0.005$.

(G) RNA-FISH of the GFP reporter mRNA (teal) from Fig. 5E in HeLa cells coexpressing MCP-mCherry-SEC61B (magenta) to visualize colocalization between the mRNA and the rough ER membrane. Representative confocal images are shown. Scale bar, 5 μm.

(H) Line profiles of the fluorescence intensities of the arrows from (G).

(I) Quantification of the experiment from (G). Two line profiles were generated for each cell. The Pearson's correlation coefficients between the reporter mRNA and the ER were determined. For MCP, $N = 26$ cells were analyzed, for MCP-SEC61B, $N = 26$ cells were analyzed. The horizontal line denotes the median and the error bars denote the 25th and 75th percentiles. Mann-Whitney test, ****, $P < 0.0001$.

(J) Schematic of a second GFP-tagged mRNA reporter that investigates the influence of subcellular mRNA localization on protein expression. Fusion of MCP to TRAPα localizes the GFP reporter mRNA to the ER membrane, whereas MCP alone localizes it to the cytosol.

(K) Confocal live cell imaging of HeLa cells expressing mC-tagged TRAPα-MCP. Scale bar, 5 μm.

(L) GFP protein expression of the reporter mRNAs from (J) coexpressing the indicated MCP-fusion constructs in HeLa cells measured by FACS. Representative histograms are shown. The histograms on the left indicate GFP-negative cell populations.

(M) Quantification of the experiment from (L). Shown is the mean ± std of four independent experiments. T-test for independent samples, ****, $P < 0.0001$.

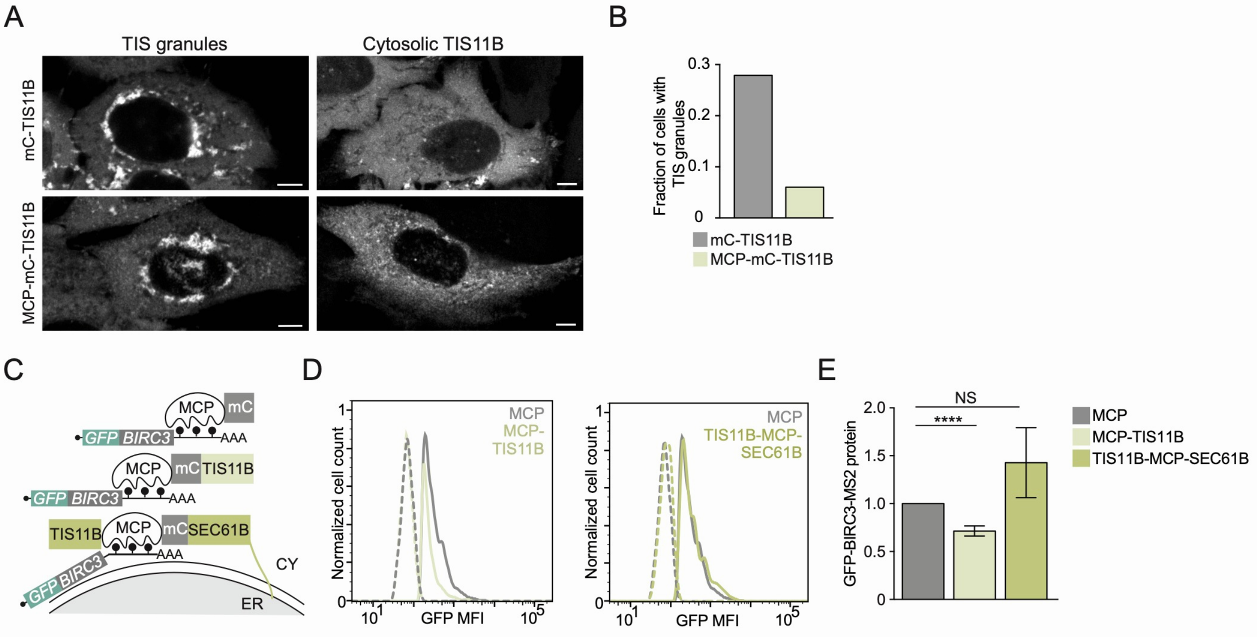

Figure S7. Redirecting mRNA localization from the cytosol to the rough ER overcomes the repressive effect of a bound RBP, related to Figure 6.

(A) Confocal live cell imaging of HeLa cells expressing the indicated constructs. Shown are representative images with TGs or cytosolic TIS11B. mC, mCherry. Scale bar, 5 μm.

(B) Quantification from (A). The fraction of HeLa cells with TGs is shown after transfection of the indicated TIS11B fusion constructs. N = 165 cells were analyzed for mCherry-TIS11B and N = 198 cells were analyzed for MCP-mCherry-TIS11B. MCP-mCherry-TIS11B largely prevents TG formation.

(C) Schematic of a second TIS11B-bound mRNA reporter that allows investigation of mRNA localization-dependent GFP expression. The coding region of the reporter is provided by BIRC3, followed by MS2 binding sites. Tethering of MCP or TIS11B localizes the mRNA reporter to the cytosol, whereas the MCP-TIS11B-SEC61B fusion localizes the mRNA reporter to the rough ER.

(D) GFP protein expression of the reporter mRNA from (C) in HeLa cells, coexpressing the indicated MCP-fusion constructs, measured by FACS. Representative histograms are shown. The histograms on the left indicate GFP-negative cell populations.

(E) Quantification of the experiment from (D). Shown is the mean ± std of four independent experiments. T-test for independent samples, ****, $P < 0.0001$, MCP vs TIS11B-SEC61B: $P = 0.058$; NS).

Table S3. Mann-Whitney statistical test values, related to Figures 1, 2, 3, 4, 5, and 7.

Related to Figure	Comparison	*Z* Score	*P* Value
1D	Unbiased vs TG	-51.9	0
1E	Unbiased vs ER	-46.1	0
1F	Unbiased vs CY	-55.5	0
1I	TG area proportion vs TG foci proportion	-6.6	5.1E-11
1I	ER area proportion vs ER foci proportion	-7.4	1.1E-6
2A	Unbiased vs TG+	-20.6	5.5E-94
2A	Unbiased vs ER+	-0.87	0.39
2A	Unbiased vs CY+	-5.0	8.9E-40
2B	Unbiased vs TG+	-11.6	3.5E-31
2B	Unbiased vs ER+	-7.4	1.5E-13
2B	Unbiased vs CY+	-5.0	5.4E-7
2C	Unbiased vs TG+	-10.6	2.5E-26
2C	Unbiased vs ER+	-4.3	1.8E-5
2C	Unbiased vs CY+	-18.0	1.0E-72
2D	Unbiased vs TG+	-3.7	2.4E-4
2D	Unbiased vs ER+	-5.3	8.5E-8
2D	Unbiased vs CY+	-8.6	6.1E-18
2E	Unbiased vs TG+	-2.3	0.024
2E	Unbiased vs ER+	-20.5	1.5E-93
2E	Unbiased vs CY+	-22.3	1.5E-110
2F	Unbiased vs TG+	-2.3	0.020
2F	Unbiased vs ER+	-13.1	3.9E-39
2F	Unbiased vs CY+	-22.9	1.2E-115
2G	Unbiased vs TG+	-2.7	0.007
2G	Unbiased vs ER+	-0.193	0.85
2G	Unbiased vs CY+	-15.2	2.6E-52
2H	Unbiased vs TG+	-17.4	1.7E-67
2H	Unbiased vs ER+	-3.7	2.5E-4
2H	Unbiased vs CY+	-10.0	1.9E-23
3F	No RBP vs TIS11B	-12.3	8.7E-35
3F	No RBP vs LM-RBP	-0.481	0.630
3F	No RBP vs TIS11B	-10.2	2.4E-24
3F	No RBP vs LM-RBP	-3.5	4.5E-4
3F	No RBP vs TIS11B	-9.1	5.9E-20
3F	No RBP vs LM-RBP	-5.2	52.5E-7
3F	No RBP vs TIS11B	-5.9	4.2E-9
3F	No RBP vs LM-RBP	-6.2	7.0E-10
3G	No RBP vs TIA1/L1	-3.9	1.1E-4
3G	No RBP vs LM-RBP	-3.4	0.001
3G	No RBP vs TIA1/L1	-4.1	3.7E-5
3G	No RBP vs LM-RBP	-3.1	0.002
3G	No RBP vs TIA1/L1	-3.3	0.001
3G	No RBP vs LM-RBP	-3.3	0.001
3G	No RBP vs TIA1/L1	-3.4	0.001
3G	No RBP vs LM-RBP	-2.2	0.027
3H	No RBP vs LM-RBP	-6.3	3.2E-10
3H	No RBP vs TIS11B	-5.7	1.4E-8
3H	No RBP vs LM-RBP	-7.3	3.8E-13
3H	No RBP vs TIS11B	-6.9	5.9E-12
3H	No RBP vs LM-RBP	-6.1	1.5E-9

3H	No RBP vs TIS11B	-7.8	6.1E-15
3H	No RBP vs LM-RBP	-3.1	0.002
3H	No RBP vs TIS11B	-4.5	5.6E-6
4C	No change vs Up in ER	-2.6	0.009
4C	No change vs Up in CY	-1.4	0.148
4C	Up in ER vs Up in CY	-3.1	0.002
4D	No change vs Up in ER	-2.0	0.048
4D	No change vs Up in CY	-3.9	9.2E-5
4D	Up in ER vs Up in CY	-1.1	0.281
5A	No RBP vs TIS11B	-1.0	0.296
5A	No RBP vs TIA1/L1	-7.9	2.4E-15
5A	TIS11B vs TIA1/L1	-7.9	2.3E-15
5O	No RBP vs TIA1/L1, unbiased	-3.8	1.7E-4
5O	No RBP vs TIA1/L1, TG+	-2.0	0.044
5O	No RBP vs TIA1/L1, ER+	-6.3	2.8E-10
5O	No RBP vs TIA1/L1, CY+	-2.7	0.008
7I	Down vs Up in iKO, TG	-7.9	2.1E-15
7I	Down vs Up in iKO, CY	-7.0	3.3E-12
S1H	ER+ vs CY+, ERM	-5.6	1.6E-8
S1H	ER+ vs CY+, Cytosol	-4.1	3.5E-5
S1K	TG+ vs ER+, Granule proportion in LoRNA	-3.1	0.002
S1K	TG+ vs CY+, Granule proportion in LoRNA	-18.4	2.7E-75
S1K	ER+ vs CY+, Granule proportion in LoRNA	-14.7	5.9E-49
S1K	TG+ vs ER+, Membrane proportion in LoRNA	-20.0	1.5E-88
S1K	TG+ vs CY+, Membrane proportion in LoRNA	-20.3	1.4E-91
S1K	ER+ vs CY+, Membrane proportion in LoRNA	-31.1	5.8E-213
S1K	TG+ vs ER+, Cytosol proportion in LoRNA	-7.4	1.4E-13
S1K	TG+ vs CY+, Cytosol proportion in LoRNA	-25.1	3.1E-139
S1K	ER+ vs CY+, Cytosol proportion in LoRNA	-27.0	3.8E-160
S2H	TG area proportion vs TG foci proportion, *BAG3*	-5.1	2.5E-7
S2H	TG area proportion vs TG foci proportion, *DUSP1*	--4.4	7.9E-6
S2H	TG area proportion vs TG foci proportion, *DNAJB1*	-3.2	0.001
S2H	TG area proportion vs TG foci proportion, *PLA2GA4*	-2.6	0.01
S2H	TG area proportion vs TG foci proportion, *IDH1*	-1.7	0.088
S2H	TG area proportion vs TG foci proportion, *ALDH18A1*	-0.034	0.973
S2H	TG area proportion vs TG foci proportion, *TES*	-2.7	0.007
S2H	TG area proportion vs TG foci proportion, *ACTN4*	-1.9	0.059
S2H	ER area proportion vs ER foci proportion, *BAG3*	-1.6	0.103
S2H	ER area proportion vs ER foci proportion, *DUSP1*	-0.916	0.437
S2H	ER area proportion vs ER foci proportion, *DNAJB1*	-0.377	0.706
S2H	ER area proportion vs ER foci proportion, *PLA2GA4*	-3.8	1.6E-4
S2H	ER area proportion vs ER foci proportion, *IDH1*	-3.3	0.001
S2H	ER area proportion vs ER foci proportion, *ALDH18A1*	-2.5	0.011
S2H	ER area proportion vs ER foci proportion, *TES*	-2.2	0.028
S2H	ER area proportion vs ER foci proportion, *ACTN4*	-1.6	0.104
S4A	Unbiased vs lowest TG+	-9.2	2.9E-20
S4A	Unbiased vs lowest ER+	-1.1	0.252
S4A	Unbiased vs lowest CY+	-6.7	2.9E-11
S4A	Lowest TG+ vs lowest ER+	-6.9	3.8E-12
S4A	Lowest TG+ vs lowest CY+	-11.5	9.9E-31
S4A	Lowest ER+ vs lowest CY+	-3.3	0.001
S4B	Unbiased vs lowest TG+	-6.6	3.3E-11

S4B	Unbiased vs lowest ER+	-4.0	5.2E-5
S4B	Unbiased vs lowest CY+	-2.7	0.006
S4B	Lowest TG+ vs lowest ER+	-7.4	9.5E-14
S4B	Lowest TG+ vs lowest CY+	-7.2	4.4E-13
S4B	Lowest ER+ vs lowest CY+	-1.4	0.148
S4C	Unbiased vs TG+	-20.6	5.9E-94
S4C	Unbiased vs ER+	-2.6	0.008
S4C	Unbiased vs CY+	-13.2	1.1E-39
S4D	Unbiased vs lowest TG+	-5.1	4.4E-7
S4D	Unbiased vs lowest ER+	-3.0	0.003
S4D	Unbiased vs lowest CY+	-8.7	3.2E-18
S4D	Lowest TG+ vs lowest ER+	-1.1	0.264
S4D	Lowest TG+ vs lowest CY+	-9.8	6.6E-23
S4D	Lowest ER+ vs lowest CY+	-7.9	2.0E-15
S4E	Unbiased vs lowest TG+	-0.79	0.43
S4E	Unbiased vs lowest ER+	-3.9	9.5E-5
S4E	Unbiased vs lowest CY+	-4.5	7.0E-6
S4E	Lowest TG+ vs lowest ER+	-3.4	0.001
S4E	Lowest TG+ vs lowest CY+	-2.5	0.012
S4E	Lowest ER+ vs lowest CY+	-6.4	1.5E-10
S4F	Unbiased vs lowest TG+	-0.689	0.491
S4F	Unbiased vs lowest ER+	-11.3	1.4E-29
S4F	Unbiased vs lowest CY+	-10.6	1.9E-26
S4F	Lowest TG+ vs lowest ER+	-9.1	1.0E-19
S4F	Lowest TG+ vs lowest CY+	-8.8	1.3E-18
S4F	Lowest ER+ vs lowest CY+	-15.3	2.4E-53
S4G	Unbiased vs lowest TG+	-1.0	0.308
S4G	Unbiased vs lowest ER+	-6.7	1.6E-11
S4G	Unbiased vs lowest CY+	-10.3	4.6E-15
S4G	Lowest TG+ vs lowest ER+	-4.9	1.1E-6
S4G	Lowest TG+ vs lowest CY+	-8.7	5.2E-18
S4G	Lowest ER+ vs lowest CY+	-11.9	1.9E-32
S4H	Unbiased vs lowest TG+	-2.2	0.026
S4H	Unbiased vs lowest ER+	-0.782	0.434
S4H	Unbiased vs lowest CY+	-6.1	1.2E-9
S4H	Lowest TG+ vs lowest ER+	-0.926	0.354
S4H	Lowest TG+ vs lowest CY+	-6.3	2.8E-10
S4H	Lowest ER+ vs lowest CY+	-4.7	2.8E-6
S4I	Unbiased vs lowest TG+	-7.7	1.2E-14
S4I	Unbiased vs lowest ER+	-0.953	0.34
S4I	Unbiased vs lowest CY+	-4.0	6.8E-5
S4I	Lowest TG+ vs lowest ER+	-5.3	1.3E-7
S4I	Lowest TG+ vs lowest CY+	-8.2	1.9E-18
S4I	Lowest ER+ vs lowest CY+	-3.7	2.5E-4
S4J	Unbiased vs TG+	-10.9	1.1E-22
S4J	Unbiased vs ER+	-18.8	8.4E-79
S4J	Unbiased vs CY+	-14.8	1.7E-49
S4K	Unbiased vs lowest TG+	-4.6	3.8E-6
S4K	Unbiased vs lowest ER+	-11.0	5.8E-28
S4K	Unbiased vs lowest CY+	-7.0	3.2E-12
S4K	Lowest TG+ vs lowest ER+	-11.5	7.6E-31
S4K	Lowest TG+ vs lowest CY+	-1.4	0.62
S4K	Lowest ER+ vs lowest CY+	-13.2	5.1E-40
